# A retrospective study on machine learning-assisted stroke recognition for medical helpline calls

**DOI:** 10.1038/s41746-023-00980-y

**Published:** 2023-12-19

**Authors:** Jonathan Wenstrup, Jakob Drachmann Havtorn, Lasse Borgholt, Stig Nikolaj Blomberg, Lars Maaloe, Michael R. Sayre, Hanne Christensen, Christina Kruuse, Helle Collatz Christensen

**Affiliations:** 1 Department of Neurology, Copenhagen University Hospital, Herlev and Gentofte, Borgmester Ib Juuls Vej 1, 2730 Herlev, Denmark; 2 Copenhagen Emergency Medical Services, Telegrafvej 5, 2750 Ballerup, Denmark; 3 Corti, Store Strandstræde 21, 1255 Copenhagen, Denmark; 4 Department of Applied Mathematics and Computer Science, Technical University of Denmark, Richard Petersens Plads, 321, 223, 2800 Kgs Lyngby, Denmark; 5 Department of Electronic Systems, Aalborg University, Fredrik Bajers Vej 7K, 9220 Aalborg Ø, Denmark; 6 Pioneer Centre for Artificial Intelligence, Øster Voldgade 3, 1350 Copenhagen, Denmark; 7 Prehospital Centre Region Zealand, Ringstedgade 61, 4700 Næstved, Denmark; 8 Department of Emergency Medicine, University of Washington, 325 9th Ave, Box 359727, Seattle, WA 98104 USA; 9 Department of Neurology, Copenhagen University Hospital, Bispebjerg, Bispebjerg Bakke 23, 2400 Copenhagen, NV Denmark; 10 University of Copenhagen, Department of Clinical Medicine, Blegdamsvej 3B, 2200 Copenhagen, Denmark

**Keywords:** Health services, Databases, Epidemiology

## Abstract

Advanced stroke treatment is time-dependent and, therefore, relies on recognition by call-takers at prehospital telehealth services to ensure fast hospitalisation. This study aims to develop and assess the potential of machine learning in improving prehospital stroke recognition during medical helpline calls. We used calls from 1 January 2015 to 31 December 2020 in Copenhagen to develop a machine learning-based classification pipeline. Calls from 2021 are used for testing. Calls are first transcribed using an automatic speech recognition model and then categorised as stroke or non-stroke using a text classification model. Call-takers achieve a sensitivity of 52.7% (95% confidence interval 49.2–56.4%) with a positive predictive value (PPV) of 17.1% (15.5–18.6%). The machine learning framework performs significantly better (*p* < 0.0001) with a sensitivity of 63.0% (62.0–64.1%) and a PPV of 24.9% (24.3–25.5%). Thus, a machine learning framework for recognising stroke in prehospital medical helpline calls may become a supportive tool for call-takers, aiding in early and accurate stroke recognition.

## Introduction

Stroke is a leading cause of disability and death worldwide^1–3^. Effective treatment is time-sensitive, and an optimal outcome is more likely when treatment is administered within the first four and a half hours from stroke onset^4,5^. The gateway to ambulance transport and hospital admittance is through prehospital telehealth services, including emergency medical call centres, nurse advice call lines, and out-of-hours health services. In the prehospital setting, the use of mobile stroke units has made it possible to deliver advanced treatment faster^6,7^. As the mobile stroke unit is only dispatched to patients with a suspected stroke, the impact of the mobile stroke unit is directly influenced by accurate call-taker recognition of stroke^6,7^. Call-takers who can rapidly and accurately recognise stroke are therefore crucial in facilitating prompt care in both prehospital and in-hospital settings.

Despite initiatives to improve stroke recognition^8,9^, approximately half of all patients with stroke do not receive the correct triage for their condition from call-takers^10–12^. Most initiatives aim to improve stroke recognition by call-takers via introducing more specific assessment tools^8,9^ or providing specialised training^13^. Recent advances in machine learning technology might be applied to improve stroke recognition without requiring changes to the triaging approach, and machine learning-aided identification of stroke has been suggested as a means of improving mobile stroke unit effectiveness^7^. Real-time feedback from a machine learning model can improve the recognition of out-of-hospital cardiac arrest^14,15^. Therefore, this study aimed to develop and assess the potential of machine learning in improving prehospital stroke recognition during medical helpline calls.

In this study, we use call recordings and registry data from the Copenhagen Emergency Medical Services (CEMS) and the Danish Stroke Registry (DanStroke) from 2015 to 2020. We obtained call recordings from two call lines: the 1-1-2 emergency line and the medical helpline 1813 (MH-1813). We then fit a machine learning framework to classify medical helpline calls as stroke or non-stroke. Calls are first transcribed using an automatic speech recognition model and then categorised by a text classification model trained as an ensemble of five individual models. We compare the performance of the model with that of call-takers using MH-1813 data from 2021.

## Results

### Population characteristics

Calls to the MH-1813 were divided into training, validation, and test subsets, and calls to the emergency line 1-1-2 were only used as supplementary training data (Table 1). Calls from the test year (2021) that were not associated with a diagnostic category code, which we used to evaluate call-taker performance, were separated from our primary test set, but still included to assess potential bias in this group of calls (2021 w/o category, Table 1). The 1-1-2 training data differed from the MH-1813 data regarding age, male/female ratio, and stroke prevalence (Table 1). Therefore, we performed an ablation study where 1-1-2 data were not used for training to assess whether this difference negatively impacted model performance. The training, validation, and test subsets of the MH-1813 data had similar characteristics, whereas the 2021 data without diagnostic categories differed in age and sex.

**Table 1:** Population characteristics for each data subset.

	Training (1-1-2)	Training (MH-1813)	Validation	Test	2021 w/o category
All calls					
Num. calls	155,696	1,391,301	155,825	344,030	231,009
Female	74,640 (47.94%)	792,783 (56.98%)	86,959 (55.81%)	190,974 (55.51%)	134,324 (58.14%)
Male	79,564 (51.10%)	596,760 (42.89%)	68,866 (44.19%)	153,050 (44.49%)	96,258 (41.67%)
65+ years	72,930 (46.84%)	335,146 (24.09%)	30,313 (19.45%)	65,652 (19.08%)	81,488 (35.27%)
Age (mean ± std.)	59.47 ± 21.24	47.12 ± 21.38	44.63 ± 20.08	44.31 ± 20.10	50.36 ± 22.77
Stroke calls					
Num. calls	3899	3471	360	757	679
Female	1784 (45.76%)	1654 (47.65%)	161 (44.72%)	349 (46.10%)	366 (53.90%)
Male	2115 (54.24%)	1815 (52.29%)	199 (55.28%)	408 (53.90%)	313 (46.10%)
65+ years	2968 (76.12%)	2421 (69.75%)	250 (69.44%)	555 (73.32%)	567 (83.51%)
Age (mean ± std.)	72.91 ± 12.77	70.68 ± 13.85	70.93 ± 13.83	71.51 ± 13.41	73.41 ± 14.11
Non-stroke calls					
Num. calls	151,797	1,387,830	155,465	343,273	230,330
Female	72,856 (48.00%)	791,129 (57.00%)	86,798 (55.83%)	190,625 (55.53%)	133,958 (58.16%)
Male	77,449 (51.02%)	594,945 (42.87%)	68,667 (44.17%)	152,642 (44.47%)	95,945 (41.66%)
65+ years	69,962 (46.09%)	332,725 (23.97%)	30,063 (19.34%)	65,097 (18.96%)	80,921 (35.13%)
Age (mean ± std.)	59.12 ± 21.30	47.06 ± 21.36	44.57 ± 20.05	44.25 ± 20.08	50.29 ± 22.76

### Main results

The classification model outperformed the call-takers (Table 2), with significant differences in all metrics (*p* < 0.0001, paired approximate permutation test). Excluding the 1-1-2 call line training data significantly degraded the model’s performance (*p* < 0.0001, paired approximate permutation test), despite the domain mismatch with the MH-1813 call line test data. The performance on the 2021 calls without a diagnostic category was significantly worse than that of the test set regarding F1-score, sensitivity, false positive rate (FPR), and false omission rate (FOR) (*p* < 0.0001, independent approximate permutation test). The difference in positive predictive value (PPV) was not significant (*p* = 0.298, independent approximate permutation test).

**Table 2:** Overall performance on MH-1813 test data, performance without 1-1-2 training data, and performance on data from 2021 without diagnostic categories, as well as performance on MH-1813 based on demographic subgroups (age/sex) [mean (95% CI)].

	F1-score [%] ↑	Sensitivity [%] ↑	PPV [%] ↑	FOR [%] ↓ (1 - NPV)	FPR [%] ↓ (1 - specificity)
Overall					
Call-takers	25.8 (23.7–27.9)	52.7 (49.2–56.4)	17.1 (15.5–18.6)	0.105 (0.094–0.116)	0.565 (0.539–0.590)
Model	35.7 (35.0–36.4)	63.0 (62.0–64.1)	24.9 (24.3–25.5)	0.082 (0.079–0.085)	0.419 (0.413–0.426)
w/o 1-1-2 training data					
Model	32.4 (31.8–33.1)	60.4 (59.3–61.4)	22.2 (21.6–22.7)	0.088 (0.085–0.091)	0.467 (0.460–0.474)
2021 test data w/o category					
Model	32.6 (31.9–33.4)	48.3 (47.2–49.4)	24.7 (23.9–25.3)	0.153 (0.148–0.158)	0.435 (0.427–0.443)
8–64 years					
Call-takers	15.9 (13.1–18.5)	50.5 (43.6–57.2)	9.40 (7.61–11.18)	0.036 (0.028–0.043)	0.353 (0.331–0.375)
Model	22.9 (21.8–24.0)	54.1 (52.1–56.3)	14.5 (13.8–15.3)	0.033 (0.031–0.035)	0.231 (0.226–0.236)
65+ years					
Call-takers	32.9 (30.1–35.7)	53.5 (49.4–57.6)	23.7 (21.4–26.0)	0.401 (0.352–0.449)	1.467 (1.373–1.560)
Model	42.8 (41.9–43.7)	66.3 (65.1–67.5)	31.6 (30.8–32.4)	0.290 (0.278–0.303)	1.224 (1.198–1.249)
Male					
Call-takers	30.2 (27.2–33.3)	53.9 (49.1–58.9)	21.0 (18.5–23.5)	0.124 (0.105–0.141)	0.542 (0.506–0.580)
Model	39.0 (38.0–40.1)	63.7 (62.3–65.2)	28.1 (27.3–29.0)	0.097 (0.093–0.102)	0.435 (0.425–0.445)
Female					
Call-takers	21.9 (19.1–24.6)	51.3 (46.0–56.6)	13.9 (12.0–15.8)	0.090 (0.076–0.103)	0.582 (0.547–0.616)
Model	32.4 (31.4–33.4)	62.3 (60.7–63.8)	21.9 (21.1–22.7)	0.069 (0.066–0.073)	0.407 (0.399–0.416)

*NPV* negative predictive value, *PPV* positive predictive value, *FOR* false omission rate, *FPR* false positive rate, *CI* confidence interval.

**Fig. 1:**
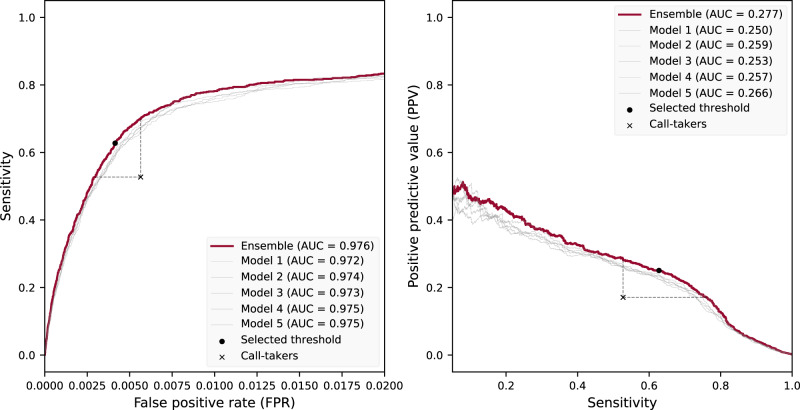
Receiver operator characteristic (ROC) curve and PPV-sensitivity curve. Left is the ROC curve, and right is the PPV-sensitivity curve (precision-recall curve). Models 1–5 are the individual models that make up the ensemble model.

**Fig. 2:**
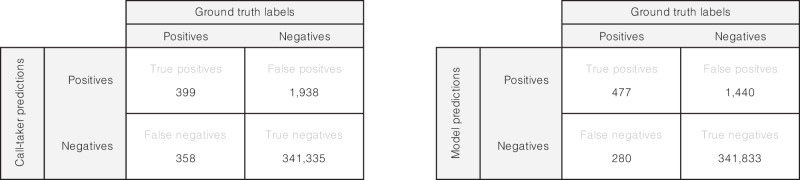
Prediction confusion matrices. Confusion matrices of predictions for call-takers and the model on the test set. Numbers for the model are given as the rounded mean over eleven runs.

The receiver operating characteristic (ROC) curve (Fig. 1, left) illustrates the potential to increase the sensitivity while maintaining an FPR lower than or equal to that of the call-takers. Similarly, the PPV-sensitivity curve (Fig. 1, right) demonstrates that sensitivity can be improved while retaining a PPV higher than that of the call-takers. The framework can thus be tuned to a sensitivity of around 73%, while still having a higher positive predictive value than the human call-taker (Fig. 1, right). The ensemble model outperformed the individual models regardless of the threshold, except for one that exhibited a slightly better sensitivity at a high FPR exceeding 1.5%. The confusion matrices (Fig. 2) illustrate the performance differences in absolute numbers, with the model exhibiting more true positives and fewer false positives than the call-takers.

### Sex and age

The model and call-takers exhibited significantly higher PPV and F1 score in men than in women (*p* < 0.0001, independent approximate permutation test) (Table 2). The model significantly outperformed the call-takers on all metrics for each sex (*p* < 0.0001, paired approximate permutation test).

The model performed significantly better in the 65+ group than in the 18–64 year group regarding sensitivity, PPV, and F1-score (*p* < 0.0001, independent approximate permutation test). Similarly, the call-takers performed significantly better in the 65+ group than in the 18–64 group regarding PPV and F1 score (*p* < 0.0001, independent approximate permutation test). Finally, the model significantly outperformed the call-takers on all metrics in both age groups (*p* < 0.0001, paired approximate permutation test).

### Model explainability

We performed an occlusion analysis to evaluate the importance of individual words for both positive and negative classifier predictions (Table 3). Among the words with a positive rank score, several words are synonymous with stroke, such as ‘blood clot’, ‘haemorrhagic stroke’, and ‘stroke’. Ambulances are rarely dispatched because the MH-1813 is not intended for emergencies. Therefore, a word like ‘ambulance’ may also be a strong indicator of call-taker recognition, which the model has learned to mimic. Additionally, most of the remaining words can be linked to stroke-related symptoms such as ‘double vision’, ‘difficulties speaking’, and ‘hangs’. Particularly, words describing the side of the body where symptoms occur ranked high (such as ‘left’, ‘right’, and ‘side’). Finally, some words were also related to the sudden onset of symptoms (including ‘suddenly’ and ‘minutes’).

**Table 3:** English translation of words with the largest positive and negative ranking score in calls predicted as stroke and non-stroke, respectively.

	Stroke predictions	*D* = 1897	Non-stroke predictions	*D* = 342,133
	Word, *w (translated)*	Occurrences, *D*^*(w)*^	Word, *w (translated)*	Occurrences, *D*^*(w)*^
1.	Ambulance	1680	Tetanus	4378
2.	Blood clot	895	Pregnant	8749
3.	Left	1108	Cut	7592
4.	Right	1050	Bandage	4561
5.	Double vision	84	Amager (a location)	23,776
6.	The words	344	O’clock	94,436
7.	Suddenly	783	The emergency room	42,809
8.	Arm	709	The police	2903
9.	Side	1,139	Swollen	60,559
10.	Stroke	117	Over-the-counter (OTC)	4641
11.	Double	113	The neck	30,151
12.	Control	134	Fever	112,586
13.	Call	39	Prescription	5450
14.	Numb	94	Centimetre	12,026
15.	Minutes	763	The knee	8875
16.	Difficulties speaking	44	The pharmacy	10,085
17.	Haemorrhagic stroke	133	The stomach	42,105
18.	Hand	297	Psychiatric	3688
19.	The ambulance	521	Pneumonia	7597
20.	Slurred speech	58	Stomach pain	10,551
21.	Blood clots	224	Stool	19,155
22.	Fast	663	The ribs	3928
23.	Express	44	Bleed	10,501
24.	Blood thinner	259	Bleeding	24,313
25.	Incoherent	15	Ribs	2941
26.	Lopsided	211	Broken	19,415
27.	Reduced	528	Inflammation	10,050
28.	Hangs	628	Common cold	8127
29.	Transient	48	Morning *or* morrow	78,558
30.	Not making sense	14	Swelling	17,762

For this analysis, we used the model with the median F1-score out of 11 randomly seeded runs.

Among the words with a negative rank score, most were strong indicators for specific conditions, symptoms, or body parts that are unrelated to stroke (such as ‘tetanus’, ‘pregnant’, ‘swollen’, ‘fever’, and ‘the knee’). Another group of words used to describe aspects of treatment that are unlikely to be addressed in a stroke call included ‘prescription’, ‘bandage’, and ‘OTC’. Finally, a small group of words described institutions that are not commonly involved in stroke treatment (such as ‘psychiatric’, ‘the emergency room’, and ‘the police’).

## Discussion

Our results showed that a machine learning framework could substantially improve stroke recognition in medical helpline calls compared to solely relying on human call-takers. This improvement was observed across all performance metrics and for basic patient demographics (*age* and *sex*). Our occlusion analysis revealed that the model relied on the relevant predictive features associated with call-taker triaging, patient symptoms, and treatment.

This study does not imply that a machine-learning model can replace medical call-takers. The effectiveness of the model is fully reliant on the conversation between the call-taker and caller and the call-taker’s ability to skillfully triage the patient. Instead, the model should be used as a supportive tool for call-takers in the decision-making process, contributing to a higher recognition of patients with stroke and potentially boosting the confidence of call-takers in their decisions. A similar machine learning model designed to predict cardiac arrest was tested in a randomised controlled trial (RCT) at CEMS^15^. The results highlighted the necessity of incorporating input from call-takers. The machine learning model for cardiac arrest has subsequently been implemented in daily practice at CEMS, in a setup similar to the one presented in our study. However, the implementation of our framework requires further investigation. The relative performance gap between call-takers and the model was larger in our study than in the cardiac arrest study^15^, which may affect the results of a potential RCT.

To support future work and discussions beyond the scope of this study, the supplementary material includes the results of a simulation of a live implementation where call-takers are assumed to follow a set of fixed rules based on the output of the machine learning framework (Supplementary Table 9). For instance, in one simulation call-takers are assumed to change any stroke negative to a positive, if the model predicts a positive. While the results of the simulation are encouraging, it is important to stress that it is not practically feasible to use a fixed rule set to overrule the call-taker. These results should only be seen as a preliminary indicator of a potential RCT. In practice, a nuanced set of guidelines should be developed over several iterations of implementation and testing.

The performance gap between the model and call-takers could be explained by the rarity of stroke calls to MH-1813 (0.250% of all calls in 2021), which might affect call-taker awareness of stroke as a possible cause of certain symptoms. Additionally, certain stroke symptoms are so rare that some call-takers may never encounter them, increasing the risk of false negatives. The model was trained on more calls than any single call-taker would handle in a lifetime, enabling it to recognise even rare descriptors of stroke. The model is specifically trained to recognise strokes and exclusively learns from actual stroke descriptions, unlike call-takers, who are trained with generalised teaching materials to triage many different conditions. Therefore, call-takers may not have received specific training for patients with stroke and may never have encountered them.

The model performed significantly better on men than on women. This could be attributed to several factors. First, the model may have learned to mimic call-takers with the same bias. Second, women may experience different and more challenging-to-identify symptoms than men^16,17^. Third, a higher prevalence of male patients with stroke was observed in the training data. Despite these potential sources of bias, the model exhibited less bias than call-takers did. That is, the relative performance improvements were higher for women than for men. This bias could be further reduced using advanced data augmentation and balanced data when training a machine learning model. However, such measures may degrade overall performance.

The improved sensitivity and PPV in the 65+ years group may be explained by a higher prior probability of stroke for older patients and stronger evidence from the patient’s medical history. The relatively high FOR and FPR for the 65+ group is likely to be a result of the much higher prevalence of stroke cases compared to the 18–64-year-olds (0.85% vs. 0.07%). We did not have data to estimate potential bias related to race, ethnicity, language, accent, or dialects. Previous studies on speech recognition for call centres have indeed found that non-native speakers had a higher rate of transcription errors^18^. Since our model was trained on a representative—and therefore unbalanced—sample, we expect it to behave similarly. Future research should look to address these shortcomings, for example, by utilising self-supervised learning on massive amounts of diverse, unlabelled data covering multiple languages, accents, and dialects.

Due to European data regulations (GDPR), it was not possible to manually transcribe MH-1813 calls to train a new speech recognition model, so we had to rely on an existing solution. This also meant that we could not evaluate the word error rate (WER) of the model. Instead, we used the downstream performance of the text classification model when trained in combination with different speech recognition models to choose the best option. Since the focus of this study is the ability to correctly recognise stroke, and not the performance of the speech recognition model alone, this approach is better suited. Indeed, the WER might be misleading when choosing a speech recognition model for a specific task. For instance, one model might fail to predict redundant minimal response words (e.g., “uh” and “uhm”) and make small inflection errors (e.g., “clot” instead of “clots”), which results in a relatively high WER, while another model only fails to predict rare, specialised words that are highly indicative of stroke (e.g., “haemorrhage” and “thrombolysis”), which results in a relatively low WER.

Although we believe that the proposed machine learning framework can be further improved, several alternatives have already been explored in the preliminary experimental phase. The speech recognition model we used was trained on 1-1-2 calls for a previous project^14^, and so, was specialised to a domain very similar to that of MH-1813. We also tested an open-source, multilingual model from OpenAI called Whisper^19^, but found that performance degraded slightly compared to the model trained on 1-1-2. We hypothesise that this is due to Whisper’s inability to handle the specific noise conditions and recognise words from a specialised medical vocabulary.

For text classification, we used an ensemble of multi-layer perceptrons (MLPs). We also tested convolutional, recurrent, and self-attention (i.e., Transformer) architectures. However, this did not improve performance. In addition, we tested a pre-trained self-supervised model. Although many of these models are freely available to the public, they are primarily trained on English data. Only relatively few options exist for the Danish language, none of which are specialised in the medical domain. We used a monolingual Danish BERT model, which has previously been shown to outperform a multilingual alternative from Google for Danish-named entity recognition^20^. However, this also did not result in a significant performance improvement. We hypothesise that the number of ground truth stroke positives was too small for these advanced models to learn more complex patterns than the MLP ensemble. In addition, a self-supervised model would likely benefit from being pre-trained on speech or text data from the target domain. Although training such large-scale foundation models has the potential to improve the classification model further, it is beyond the scope of this study. Thus, we chose the simpler MLP ensemble. We have included references to reviews of self-supervised learning for speech and text in the references^21,22^. Notably, it is not uncommon for small, simple models to match or outperform large, pre-trained models for text classification tasks^23^.

This study has some limitations. First, the mapping of call recordings to electronic records was incomplete due to technical limitations in the computer-aided dispatch (CAD) registry, which limited the number of calls available to us. Of note, there was no obvious pattern of bias related to the unmapped calls, and we included all calls with matching audio files, regardless of dispatcher performance. The results could potentially be improved if more calls were available for analysis. Second, calls without a call-taker-indicated diagnostic category were not included in the validation and test data because the call-taker’s performance could not be evaluated. Moreover, in exploratory analyses, the model performed worse on these calls, which might be attributed to differences in population characteristics (Table 1). Finally, the ground truth stroke labelling relied on the patient-reported time of onset being exact; however, estimating the accuracy of the timestamps in DanStroke was impossible.

In conclusion, using the largest collection of audio calls from patients with stroke to date, we developed a machine-learning framework that significantly outperformed human call-takers in stroke recognition in medical helpline calls. The framework can assist human call-takers during medical helpline calls. Ideally, this would enable a higher recognition of patients with stroke in the prehospital setting, benefiting both patient outcomes and health service resource allocation.

## Methods

### Data sources

#### Copenhagen emergency medical services (CEMS)

The CEMS is responsible for providing prehospital telehealth services in the Capital Region of Denmark, with a catchment area of 1.9 million^24^. CEMS operates two call lines: the 1-1-2 emergency line, similar to 9-1-1 in the United States, intended for acute conditions. The other is the medical helpline 1813 (MH-1813, pronounced ‘18-13’) intended for non-life-threatening conditions that cannot wait until a general practitioner is available^25^.

Call-takers for both lines, who are nurses, paramedics, or physicians, can dispatch ambulances. The condition suspected by the call-taker is categorised based on a predefined diagnostic index and stored in an electronic record using a CAD system. The CAD records are associated with the Danish civil registration number (CPR number)^26^ of the patient. The CPR number is a unique identification assigned to all Danish residents. It is used for interactions with health services and registries, enabling cross-referencing of the data sources used in this study. The call audio is recorded and stored separately from the CAD recordings using a telephone system.

#### Danish Stroke Registry (DanStroke)

All patients with a final diagnosis of stroke or transient ischaemic attack admitted to a Danish hospital within 5 days of symptom onset are recorded in the Danish Stroke Registry^27^, also known as DanStroke. This record includes the patient-reported time of onset, stroke type (haemorrhagic, ischaemic, or transient ischaemic attack), and CPR number of the patient. The diagnosis is obtained according to the national guidelines^28^, which includes cerebral imaging and full diagnostic workup by neurologists. The validity of the Danish stroke registry has been shown to be high^29^, and the number of stroke mimics is therefore minimised in our dataset.

#### Inclusion and ethics

The Danish Data Protection Agency (P-2021-475) approved this study. Danish law did not require approval from the Scientific Ethics Committee because the data were registry-based. CEMS approved the transcription of all calls made to 1-1-2 and MH-1813. All electronic records were anonymised before analysis, and the researchers did not inspect the calls manually.

### Study scope

Stroke prevalence in calls made to the MH-1813 is lower than that in calls made to 1-1-2. Patients with stroke may exhibit different symptoms and symptom severity because MH-1813 is meant for low-acuity incidents, leading to reduced recognition. In addition, MH-1813 call-takers dispatch high-priority transport less frequently, which may affect optimal treatment timing. Therefore, we focused on MH-1813 in this study.

### Stroke dataset

#### Cross-referencing data sources

From the CAD medical records, we included all calls that could be matched to a corresponding audio file for 1-1-2 and MH-1813 from 2015 to 2021 for patients older than 18. The CAD records were matched with the telephone call recordings based on the call start, call duration, and call-taker identity. Due to data incompleteness, and the way the audio data is stored, at CEMS, 2,730,199 contacts could not be matched to their corresponding audio file, however, 2,361,178 contacts were successfully matched. We found no obvious pattern in the matched and unmatched calls and we included all calls with a matching audio file. Next, a call was regarded as a case of ground truth stroke positive when the CPR number in the CAD record matched that of a DanStroke record, and the patient-reported time of onset was close to the call start time. We allowed a window of 72 hours before and 24 hours after the call starts to account for uncertainty in recording stroke onset time. We excluded calls involving subarachnoid haemorrhage cases. Finally, we considered a call to be a call-taker stroke positive when the call-taker selected the stroke diagnostic category during the call and dispatched an ambulance with the appropriate level of response^30^. To ensure that the effect of the machine learning framework was not overestimated, we excluded calls where diagnostic category had not been registered from the test set. We still reported the population characteristics and model performance of this group of calls to assess potential bias introduced by excluding them. A data-flow diagram is included in the supplementary material (Supplementary Fig. 1). The resulting dataset is the largest dataset of audio files from stroke calls collected to date.

#### Dataset splitting

We reserved all the MH-1813 calls from 2021 for testing. We used stratified sampling to divide the MH-1813 calls from 2015 to 2020 into validation and training subsets. The training subset was further split into five folds, which were used for ensemble training. The calls were stratified based on the ground truth stroke label and the presence of a diagnostic category. Calls without diagnostic categories were only included in the training set. The 1-1-2 calls were used only for training; however, calls from 2021 were discarded to avoid temporal overlap with the test period.

### Machine learning pipeline

We employed a two-step machine learning pipeline. First, a call was transcribed using the speech recognition model. Second, the transcript was used as input for the text classification model. The final output score was used to classify whether the call concerned a stroke. The pipeline is illustrated in the supplementary material (Supplementary Fig. 2).

#### Speech recognition

The call recordings from the CEMS were stored as 8-bit linear pulse-code modulated audio, sampled at 8 kHz. A call was converted into a log-Mel spectrogram before being input into the speech recognition model. This conversion is a commonly used input representation for speech-processing tasks, which facilitates the identification of linguistic content in audio signals. We used a speech recognition model with a neural network architecture^31^, consisting of two-dimensional convolutional layers^32^ and blocks of bidirectional long short-term memory layers^33^. The output is a sequence of probability distributions over characters of the Danish alphabet, which were then converted into a human-readable transcript using a greedy decoder^34^.

#### Text classification

As input for the classification model, each transcript was transformed into a fixed-size bag-of-words vector, which encoded the occurrence of word and character (n-grams) in a fixed vocabulary. The feature selection procedure is detailed in the Supplementary Methods. The model was constructed as an ensemble^35^ of five identical, independently trained models. Each consists of a stack of neural network layers commonly referred to as a multi-layer perceptron^36^. The final layer has a single scalar output and applies a sigmoid nonlinearity to produce an output score between zero and one.

#### Threshold calibration and ensembling

For each model in the ensemble, we selected the prediction threshold as the harmonic mean of the two thresholds that ensure sensitivity and PPV equal to those of the call-takers. This simplifies the comparison by ensuring a trade-off between sensitivity and PPV, similar to that of call-takers.

As the threshold differed for each model in the ensemble, computing the ensemble output score as the average output score of the individual models would not be meaningful. Instead, we first subtracted the threshold from the output score in logit space (before sigmoid nonlinearity) for each model to obtain the same threshold (0.5). Subsequently, we defined the ensemble output score as the average of the centred output scores. The exact equations are provided in the supplementary material [Supplementary Equations (1) and (2)].

#### Model training

The speech recognition model was trained on 3,811 manually transcribed random calls (173 h) from the CEMS as part of a previous project^14^. These calls exclusively originated from 1-1-2 between 2015 and 2018, ensuring no overlap with the test data used for the text classification model. The model was trained using a connectionist temporal classification objective^34^.

We trained five models for the text classification ensemble using binary cross-entropy after transcribing all calls in the dataset using the speech recognition model. One training fold was used for early stopping using the F1-score, whereas the remaining fourfold and 1-1-2 data were used for training. Thus, each model in the ensemble was trained and validated using different datasets. We ran a grid search with 96 different hyperparameter configurations and selected the ensemble model with the best F1 score for the validation set.

### Model explainability

We performed an *occlusion analysis* to better understand the predictions of the text classification model. This involved removing all instances of a given word from the input transcript to evaluate its impact on the model output. The word was removed before vectorisation, such that all word and character n-grams associated with the word were discarded. Specifically, let *z*^*(n,d,w)*^ be the logit output of model *n* in the ensemble for transcript *d* when the word *w* is occluded. For transcript *d*, we computed the word impact score *i*^*(d,w)*^ as the mean difference between the logit before and after occlusion.

1 $${i}^{(d,w)}=\frac{1}{N}{\mathop{\sum}\limits_{n=1}^{N}}\,{z}^{(n,d)}-{z}^{\left(n,d,w\right)}$$

We used the logit output to compute the impact score because the difference in sigmoid-normalised output is biased towards zero for values close to 0 or 1. To select words for inspection, we computed a ranking score, *r*^*(w)*^, as the sum of the signed squares of the impact:

2 $${r}^{(w)}=\mathop{\sum }\limits_{d=1}^{D}\,\mathrm{sgn}\left({i}^{(d,w)}\right){\left({i}^{(d,w)}\right)}^{2}$$

where sgn(·) represents the sign function. Squaring *i*^*(d, w)*^ favours rare features with a high impact over common features with a low impact.

### Statistical analysis

We report the F1-score, sensitivity, PPV, FOR (equal to 1−negative predictive value), and FPR (equal to 1−specificity). Due to the imbalanced nature of the dataset, the negative predictive value and specificity were >99% for all cases. We reported FOR and FPR instead because such large numerical values exhibit low relative variance, thereby obfuscating comparisons. Finally, we report the prediction confusion matrices, ROC curve, and PPV-sensitivity curve, commonly known as the precision-recall curve. All results are reported with up to three significant digits.

We present the results with and without 1-1-2 training data, subgroup analyses based on age (18–64/65+) and sex (male/female), and call-takers performance. We also report the model performance on calls without a diagnostic category from the test year 2021 to assess potential data bias. We tested our results for statistical significance using approximate permutation tests. We used one-sided paired approximate permutation tests for model-to-model and model-to-call-taker comparisons when done on the same subset. For comparisons across different subsets (e.g., male vs. female), we used one-sided independent approximate permutation tests. We computed 95% confidence intervals (CIs) using bootstrapping^37,38^. In our assessment, we accounted for random variation associated with model training by basing the means, tests, and CIs on the predictions of 11 randomly initialised training runs. Statistical significance was defined as a *p* value of <0.05.

We used the model with the median F1-score out of the 11 runs for the occlusion analysis. We listed the 30 words with the highest positive ranking scores for calls classified as stroke and the 30 words with the highest negative ranking scores for calls classified as non-stroke.

### Reporting summary

Further information on research design is available in the Nature Research Reporting Summary linked to this article.

### Supplementary information

Supplementary

Reporting Summary

## Data Availability

The datasets used to evaluate call-taker performance and to train and evaluate the machine learning framework are legally restricted by Danish patient privacy and secrecy laws and are, therefore, not publicly available. The data can be made available from the publication date but requires a Data Access Agreement, which is examined and approved by the ethics committees that approved this research^39^. For the same reason, the machine learning framework trained in this study is not publicly available; however, instructions on how to train it are included in the main manuscript and the Supplementary Methods.

## Code availability

The source code can be shared using a Creative Commons NC-ND 4.0, an international licence, upon reasonable written request to the corresponding author, and requires a data use agreement.

## References

[1] Feigin VL (2021). Global, regional, and national burden of stroke and its risk factors, 1990-2019: a systematic analysis for the Global Burden of Disease Study 2019. Lancet Neurol..

[2] Kyu HH (2018). Global, regional, and national disability-adjusted life-years (DALYs) for 359 diseases and injuries and healthy life expectancy (HALE) for 195 countries and territories, 1990-2017: a systematic analysis for the Global Burden of Disease Study 2017. Lancet.

[3] Katan M, Luft A (2018). Global burden of stroke. Semin. Neurol..

[4] Berge E (2021). European Stroke Organisation (ESO) guidelines on intravenous thrombolysis for acute ischaemic stroke. Eur. Stroke J..

[5] Turc G (2019). European Stroke Organisation (ESO) - European Society for Minimally Invasive Neurological Therapy (ESMINT) guidelines on mechanical thrombectomy in acute ischemic stroke. J. Neurointerv. Surg..

[6] Hariharan P (2022). Mobile stroke units: current evidence and impact. Curr. Neurol. Neurosci. Rep..

[7] Navi BB (2022). Mobile stroke units: evidence, gaps, and next steps. Stroke.

[8] Krebes S (2012). Development and validation of a dispatcher identification algorithm for stroke emergencies. Stroke.

[9] Govindarajan P (2012). Feasibility study to assess the use of the Cincinnati stroke scale by emergency medical dispatchers: a pilot study. Emerg. Med. J..

[10] Oostema JA (2016). Dispatcher stroke recognition using a stroke screening tool: a systematic review. Cerebrovasc. Dis..

[11] Viereck S. et al. Medical dispatchers recognise substantial amount of acute stroke during emergency calls. *Scand. J. Trauma Resusc. Emerg. Med.*10.1186/S13049-016-0277-5 (2016). 10.1186/s13049-016-0277-5 PMC4936322 27388490

[12] Bohm K., Kurland L. The accuracy of medical dispatch - a systematic review. *Scand. J. Trauma Resusc. Emerg. Med.*10.1186/S13049-018-0528-8 (2018). 10.1186/s13049-018-0528-8 PMC6230269 30413213

[13] Watkins C. L., et al. Training emergency services’ dispatchers to recognise stroke: an interrupted time-series analysis. *BMC Health Serv. Res.* **13**, 318 (2013) 10.1186/1472-6963-13-318 PMC3751943 23947656

[14] Blomberg SN (2019). Machine learning as a supportive tool to recognize cardiac arrest in emergency calls. Resuscitation.

[15] Blomberg S. N., et al. Effect of machine learning on dispatcher recognition of out-of-hospital cardiac arrest during calls to emergency medical services: a randomized clinical trial. *JAMA Netw. Open*. **4**, e2032320 (2021). 10.1001/jamanetworkopen.2020.32320 PMC7788469 33404620

[16] Carcel C. et al. Sex matters in stroke: a review of recent evidence on the differences between women and men. *Front. Neuroendocrinol.* **59**, 100870 (2020). 10.1016/j.yfrne.2020.100870 32882229

[17] Eddelien H. S. et al. Sex and age differences in patient-reported acute stroke symptoms. *Front. Neurol.*10.3389/FNEUR.2022.846690 (2022). 10.3389/fneur.2022.846690 PMC8978710 35386418

[18] Han K. J. et al. Deep learning-based telephony speech recognition in the wild. *In:* Proceedings of the Annual Conference of the International Speech Communication Association, INTERSPEECH https://www.isca-speech.org/archive/pdfs/interspeech_2017/han17_interspeech.pdf 1323–1327 (2017).

[19] A. Radford, et al. Robust speech recognition via large-scale weak supervision. *In:* Proceedings of the 40th International Conference on Machine Learning. 28492–28518 (2023).

[20] Hvingelby, R. et al. DaNE: a named entity resource for Danish. *In*: Proceedings of the LREC 2022 Workshop of the 1st Annual Meeting of the ELRA/ISCA Special Interest Group on Under-Resourced Languages. 4597–4604 (2020).

[21] Mohamed, A. et al. Self-supervised speech representation learning: a review. *IEEE J Sel Top Signal Process* https://arxiv.org/abs/2205.10643 (2022).

[22] Gururangan, S. et al. Don’t stop pretraining: adapt language models to domains and tasks. *In:* Proceedings of the 58th Annual Meeting of the Association for Computational Linguistics 8342–8360 (2020).

[23] Galke L (2022). Bag-of-words vs. graph vs. sequence in text classification: questioning the necessity of text-graphs and the surprising strength of a wide MLP. Assoc. Comput. Linguist..

[24] Danmarks Statistik (Statistics Denmark) (2023). FOLK1: population quarterly database. https://www.statistikbanken.dk/FOLK1A (2023).

[25] Zinger N. D. et al. Impact of integrating out-of-hours services into Emergency Medical Services Copenhagen: a descriptive study of transformational years. *Int. J. Emerg. Med.*10.1186/S12245-022-00442-4 (2022). 10.1186/s12245-022-00442-4 PMC9414103 36008756

[26] Schmidt M, Pedersen L, Sørensen HT (2014). The Danish Civil Registration System as a tool in epidemiology. Eur. J. Epidemiol..

[27] Johnsen S (2016). The Danish Stroke Registry. Clin. Epidemiol..

[28] Blauenfeldt, R., Wienecke T. National Neurologisk Behandlingsvejledning: Iskæmisk apopleksi - akut udredning og behandling, https://nnbv.dk/iskaemisk-apopleksi-akut-udredning-og-behandling/ (accessed 19 September 2023).

[29] Wildenschild C (2013). Registration of acute stroke: validity in the Danish Stroke Registry and the Danish National Registry of Patients. Clin. Epidemiol..

[30] Dansk Indeks for Akuthjælp. Landsudgaven, version 1.10—revideret april 2022., https://www.ph.rm.dk/siteassets/prahospitalet/fagfolk/dansk-indeks/dansk%0A-indeks-version-1.10---landsudgaven.pdf (accessed 27 March 2023).

[31] Borgholt L. et al. Do end-to-end speech recognition models care about context? *In*: Proceedings of the Annual Conference of the International Speech Communication, 4352–4356 https://arxiv.org/abs/2102.09928 (2020).

[32] Lecun Y., Bengio Y. Convolutional networks for images, speech, and time-series, https://nyuscholars.nyu.edu/en/publications/convolutional-networks-for-images-speech-and-time-series (1995, accessed 20 April 2023).

[33] Hochreiter S, Schmidhuber J (1997). Long short-term memory. Neural Comput..

[34] Graves A. et al. Connectionist temporal classification: labelling unsegmented sequence data with recurrent neural networks. 369–376 (2006).

[35] Hansen LK, Salamon P (1990). Neural network ensembles. IEEE Trans. Pattern Anal. Mach. Intell..

[36] Rosenblatt F (1958). The perceptron: a probabilistic model for information storage and organization in the brain. Psychol. Rev..

[37] Dwass M (1957). Modified randomization tests for nonparametric hypotheses. Ann. Math. Stat..

[38] Eden T, Yates F (1933). On the validity of Fisher’s z test when applied to an actual example of non-normal data. (With five text-figures.). J. Agric. Sci..

[39] Danish Data Protection Agency. https://www.datatilsynet.dk/english (accessed 14 November 2023).

