# Supplementary material for: A retrospective study on machine learning-assisted stroke recognition for medical helpline calls
Source: NPJ Digit Med. 2023 Dec 19;6:235. doi: 10.1038/s41746-023-00980-y (PMC10730829; doi:10.1038/s41746-023-00980-y)
Supplement: Supplementary file 1 — Supplementary [file 41746_2023_980_MOESM1_ESM.pdf]

Retrospective Study on Machine Learning-Assisted Stroke Recognition for Medical Helpline Calls

Supplementary material

SUPPLEMENTARY FIGURE 1: DATA FLOW DIAGRAM **2**

SUPPLEMENTARY METHODS: MACHINE LEARNING PIPELINE **2**

Model training *2*

Hyperparameters *2*

Ensembling details *5*

SUPPLEMENTARY METHODS: SIGNIFICANCE TESTING AND CONFIDENCE INTERVALS **5**

SUPPLEMENTARY METHODS: SOFTWARE **7**

SUPPLEMENTARY RESULTS: MODEL PERFORMANCE ACROSS DEMOGRAPHICS **7**

SUPPLEMENTARY RESULTS: MODEL WITH PATIENT AGE AND SEX AS EXPLICIT INPUTS **8**

SUPPLEMENTARY RESULTS: MODEL WITHOUT MH-1813 TRAINING DATA **10**

SUPPLEMENTARY RESULTS: DETAILED MODEL EXPLAINABILITY TABLES **12**

SUPPLEMENTARY RESULTS: FINE-TUNING OF DANISH BERT MODEL FOR STROKE RECOGNITION **14**

SUPPLEMENTARY RESULTS: SIMULATION OF A PROSPECTIVE STUDY ON 2021 DATA **15**

Method *15*

Results *15*

SUPPLEMENTARY DISCUSSION: RESEARCH IN CONTEXT **17**

Evidence before this study *17*

Added value of this study *17*

Implications of all the available evidence *17*

SUPPLEMENTARY DISCUSSION: RESEARCH IN CONTEXT SEARCH TERM RESULTS **18**

REFERENCES **25**

SUPPLEMENTARY FIGURE 1: DATA FLOW DIAGRAM

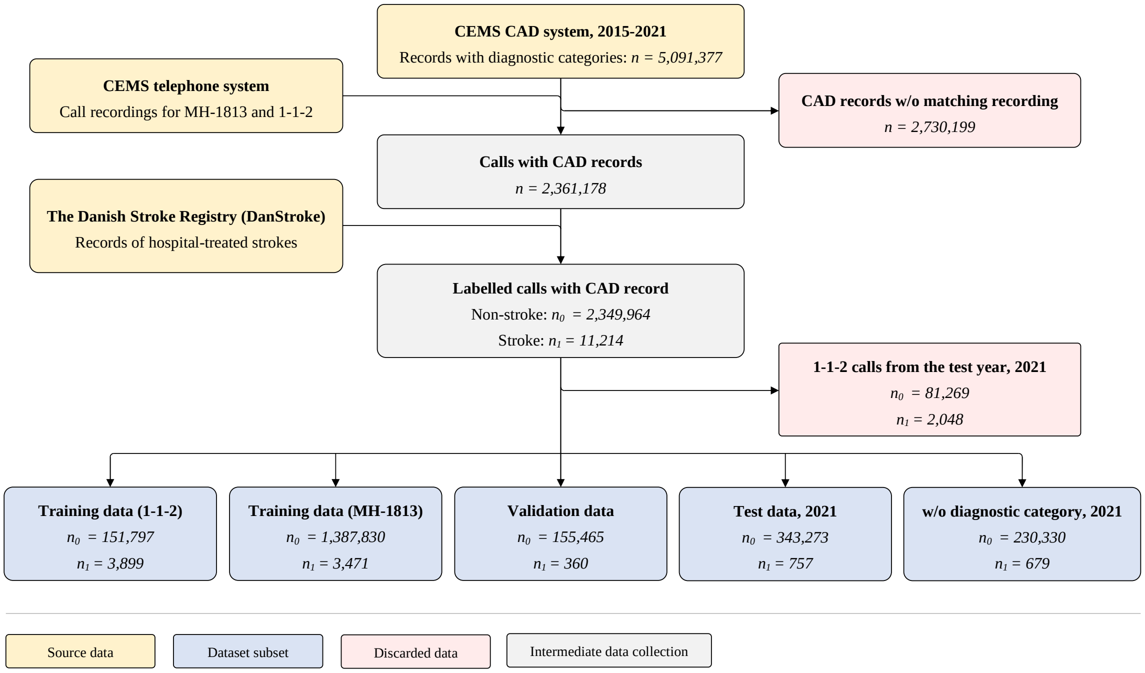

Supplementary Figure 1. Overview of data flow from the initial data sources to the final stroke dataset

SUPPLEMENTARY METHODS: MACHINE LEARNING PIPELINE

Model training

We used stochastic gradient descent on mini-batches of data to train the stroke classification model. We used the Adam (adaptive moment estimation) optimisation algorithm and ensured an equal number of stroke positives and negatives in each batch by stratifying the class labels during sampling. We saved the model parameters after each epoch if the maximum F1-score (across all possible thresholds) improved in the validation dataset. We used the latest saved parameters as the final result of the run.

Hyperparameters

The selection of hyperparameters followed a simple two-stage process using validation data (table 1). First, a manual search was conducted by running different model configurations with varying numbers of epochs, updates per epoch, batch sizes, vectoriser types, and hyperparameters. Subsequently, a structured grid search was performed to further tune a subset of these hyperparameters.

Supplementary Table 1. Overview of hyperparameters used for training the text classification models.

Name	Chosen value	Grid search range
Determined from initial heuristic, manual hyperparameter search on validation fold		
Epochs	30	-
Parameter updates per epoch	500	-
Batch size	128	-
Batch sampling	Label stratified (balanced)	-
Type of text vectoriser	Count vectorisation	-
Size of bag-of-words vector	10,000	-
Optimization algorithm	Adam (β_1=0.9, β_2=0.999)	-
Learning rate schedule	Cosine annealing from the start	-
Determined from grid search on validation fold		
Learning rate start	0.0003	{0.003, 0.0003}
Learning rate end	0.0003	{0.0003, 0.00003, 0.000003}
Model input dropout	0.50	{0.25, 0.50}
Model dropout	0.25	{0.25, 0.50}
Model configuration	[256, 128, 64, 32, 16]	{[64, 32, 16], [256, 128, 64, 32, 16]}

Bag-of-words selection

Each transcript was transformed into a fixed-size bag-of-words vector to serve as input for the classification model. These vectors encode the occurrence of words and character n-grams within a fixed vocabulary. We selected vocabulary by first computing the $\chi 2$-statistics for all word uni- and bi-grams and character three-, four-, and five-grams that occurred in more than ten training calls. We then retained the M highest-scoring word n-grams and M highest-scoring character n-grams, yielding $2M$ input features, where M represents a tuned hyperparameter. By complementing word n-grams with character n-grams, the model can use out-of-vocabulary words not included in the word n-grams and robustly represent words misspelt by the speech recogniser. The feature vector was input into the classification model (figure 2.C).

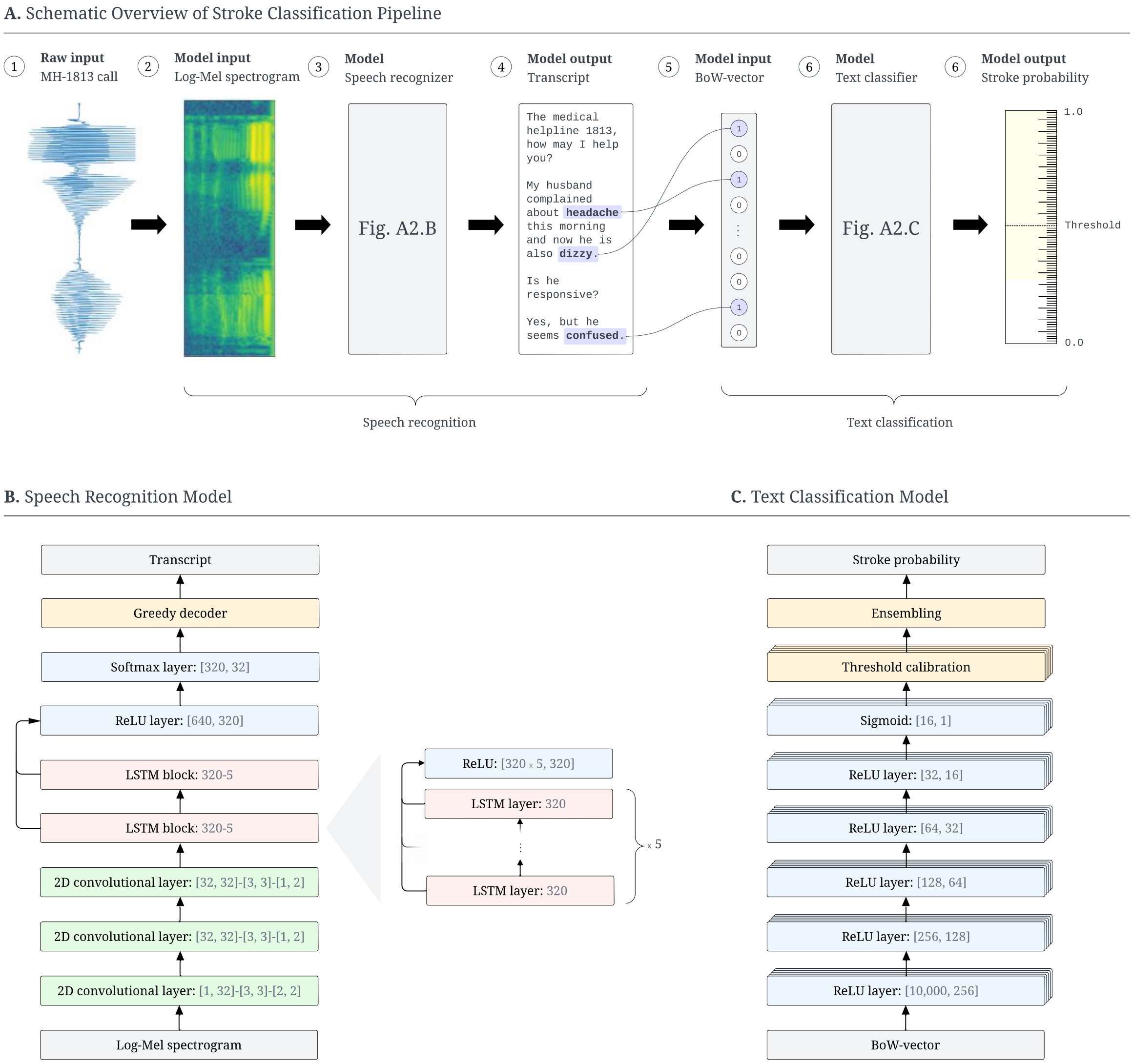

Supplementary Figure 2. Overview of machine learning pipeline. Figure 2.A presents a schematic overview of the machine learning pipeline. The individual models are broken down in figures 2.B and 2.C. The 2D convolutional layers have parameters [*input channels*, *output channels*]-[*kernel width*, *kernel height*]-[*stride width*, *stride height*]. The long short-term memory (LSTM) blocks have parameters *hidden units*-*number of layers*. The rectified linear unit (ReLU), sigmoid, and softmax layers have parameters [*input features*, *output features*]. Joining arrows indicate the concatenation of two vector sequences along the feature dimension. The full set of model hyperparameters is listed in the supplementary material.

As part of our manual hyperparameter search, we trained the models using vectorisers of different sizes. We discovered that using 5,000-word n-grams and 5,000-character *n*-grams (M=*5,000*) struck a good trade-off between size, feature quality, and model performance), yielding 10,000 bag-of-words features.

Ensembling details

A common way to combine individual classification models into an ensemble is to use a voting scheme, such as majority voting, where a combined prediction is made based on the consensus among the individual models. However, this approach makes the ensemble not have a continuous output score. This is problematic for two reasons.

The lack of a continuous output score prevents the evaluation of the model's performance across a continuous range of thresholds required for plotting the receiver operating characteristic and precision-recall curves.

The lack of a continuous output score deteriorates the quality of assessing the effect of different words on ensemble performances (see *Model explainability*).

Therefore, we used a different ensemble method, which is briefly described in the main text. Herein, we provide a mathematically rigorous definition of the proposed method.

Let $z^{(n, d)}$ be the logit output of model n for transcript d, $t^{(n)}$ the tuned logit threshold of model n, and N the number of models in the ensemble. The output score $p^{(d)}$ is then given by

$$p^{(d)} = \frac{1}{N} \sum_{n=1}^{N} \sigma(z^{(n,d)} - t^{(n)}) \ , \qquad (1)$$

where $\sigma(\cdot)$ is the sigmoid function (or standard logistic function). The final ensemble prediction $s^{(d)}$ is then simply

$$s^{(d)} = I_{p>0.5}\left(p^{(d)}\right) \ , \qquad (2)$$

where $I(\cdot)$ is the indicator function that returns 1 if the subscript condition is satisfied and 0 otherwise.

SUPPLEMENTARY METHODS: SIGNIFICANCE TESTING AND CONFIDENCE INTERVALS

We used standard methods for significance testing and computing the confidence intervals. We used approximate methods owing to data size and to maintain computational feasibility.[1,2]

We performed *paired approximate permutation tests* by pairing each observation from the first sample to a random observation from the other sample (without replacement), while keeping each observation within its original sample. This allowed us to test the significance of the observed pairings on the chosen statistics, i.e. whether a significant difference was observed in the test statistics depending on whether the call taker or model made the prediction. We used this approach to test whether

- the model performed better than the call takers on the 2021 test set,
- including 112 training data improved the model's performance on the 2021 test set.

We performed *independent approximate permutation* tests by randomly assigning observations to either of the two samples (without replacement) while maintaining any differences in sample size.

This approach allowed us to test the significance of the observed sample assignments, i.e. whether there was a significant difference in test statistics depending on whether the predictions were assigned to the model or call taker. We used this approach to test whether.

- the model performed better on the 2021 test set with diagnostic categories than on the test set without diagnostic categories,
- the model performed better on men than women on the 2021 test set,
- the model performed better on the 65+ group than on the 18–64 group on the 2021 test set,
- the call taker performed better on men than women on the 2021 test set,
- the call taker performed better on the 65+ group than on the 18–64 group on the 2021 test set.

The p-values were not exact because we used approximate permutation tests. However, owing to the large dataset size and substantial number of observations, the estimated p-values had tight confidence intervals. We reported the upper bound of the 99% confidence interval on the p-value computed as the usual binomial distribution confidence interval.

$$CI(p) = \hat{p} \pm z\sqrt{\frac{\hat{p}(1-\hat{p})}{N}}\ , \tag{3}$$

where *N* is the number of resamplings.

We computed *bootstrapped confidence intervals* for the statistics by resampling (with replacement) the predictions made by model or call taker on a relevant subgroup and recomputing the relevant statistics for each bootstrap sample. This process established a bootstrap distribution of the statistic that was then used to estimate the standard error and compute the confidence intervals. We computed confidence intervals using the bootstrap distribution percentiles. This method yielded reliable results because our dataset was large, and the bootstrap distribution was symmetrical and centred on the observed statistic. No observed bootstrap distributions differed significantly from normal distributions (according to Anderson–Darling and Shapiro–Wilk tests). Therefore, confidence intervals computed alternatively as studentised bootstrap intervals (t-intervals) did not differ substantially from percentile confidence intervals. This further validated our tests.

We used *N*=15,000 resamplings for permutation tests and N=8,192 for confidence intervals.

SUPPLEMENTARY METHODS: SOFTWARE

We used Python version 3.8.10. PyTorch version 1.12.1 + cu113 was used to train the neural network models. We used SciKit-Learn (version 1.2.2) to perform bag-of-words vectorisation. We used NumPy version 1.23.5, Pandas version 1.5.3, Matplotlib version 3.7.1, and SciPy version 1.10.1 to perform data analysis, plotting, and testing.

SUPPLEMENTARY RESULTS: MODEL PERFORMANCE ACROSS DEMOGRAPHICS

When 1-1-2 data were not used for training (table 2), the model performed significantly better in men than in women on the test set in terms of all metrics ($p < 0.0001$, paired approximate permutation test) and significantly better on the 65+ group than on the 18–64 group in terms of all metrics ($p < 0.0001$, paired approximate permutation test). As noted in the main text, both these statements were also true when 1-1-2 data were used for the training (tables 3 and 4) and that including 1-1-2 data significantly improved overall performance ($p < 0.0001$, paired approximate permutation test). Compared to the test set (table 2), model performance on the 2021 calls without diagnostic category was significantly worse in all demographic subgroups (table 3) in terms of all metrics ($p < 0.0001$, independent approximate permutation test), except for positive predictive value (PPV) for male (where $p = 0.0056$ [significant], independent approximate permutation test) and for the false positive rate (FPR) on females and individuals aged 65+ years (where $p = 0.213$ and $p = 0.362$, respectively [insignificant] , independent approximate permutation test).

Supplementary Table 2. Model performance grouped by sex and age [mean (95% CI)] when 1-1-2 training data is not used for training. NPV: negative predictive value, PPV: positive predictive value, FOR: false omission rate, CI: confidence interval.

	Female	Male	18–64 years	65+ years
F1-score [%] ↑	28.7 (27.8–29.6)	36.2 (35.3–37.2)	20.5 (19.5–21.5)	39.4 (38.6–40.3)
Sensitivity [%] ↑	58.2 (56.7–59.9)	62.1 (60.7–63.6)	53.0 (50.9–55.0)	63.0 (61.9–64.3)
PPV [%] ↑	19.0 (18.3–19.7)	25.6 (24.8–26.4)	12.7 (12.0–13.4)	28.7 (27.9–29.5)
FOR [%] ↓ (1 - specificity)	0.077 (0.073–0.080)	0.102 (0.097–0.106)	0.034 (0.032–0.036)	0.318 (0.305–0.331)
FPR [%] ↓ (1 - NPV)	0.45 (0.446–0.463)	0.483 (0.473–0.494)	0.264 (0.258–0.270)	1.335 (1.308–1.362)

Supplementary Table 3. Model performance grouped by sex and age [mean (95% CI)] on the 2021 data without diagnostic category. NPV: negative predictive value, PPV: positive predictive value, FOR: false omission rate, CI: confidence interval.

	Female	Male	18–64 years	65+ years
F1-score [%] ↑	30.4 (29.3–31.5)	35.2 (34.0–36.4)	24.3 (23.0–25.7)	36.6 (35.6–37.6)
Sensitivity [%] ↑	44.5 (42.9–46.0)	52.8 (51.1–54.4)	45.6 (43.4–47.9)	49.2 (47.9–50.5)
PPV [%] ↑	23.1 (22.2–24.1)	26.4 (25.3–27.4)	16.6 (15.5–17.5)	29.1 (28.2–30.0)
FOR [%] ↓ (1 – specificity)	0.152 (0.146–0.159)	0.155 (0.147–0.162)	0.058 (0.055–0.062)	0.372 (0.359–0.386)
FPR [%] ↓ (1 – NPV)	0.404 (0.394–0.414)	0.480 (0.467–0.494)	0.246 (0.239–0.254)	0.873 (0.852–0.894)

SUPPLEMENTARY RESULTS: MODEL WITH PATIENT AGE AND SEX AS EXPLICIT INPUTS

To assess the importance of patient age and sex for accurate model stroke recognition we have performed an experiment to test whether explicitly adding the age and sex of the patient as inputs to the model improves performance.

Specifically, we encode patient sex as two binary numbers which we concatenate to the bag-of-words input representation. The first has the value of 1 if the patient is female, and the second has the value of 1 if the patient is male. Neither is 1 if the patient's sex is unknown/undisclosed. Similarly, we include patient age as a 15-dimensional one-hot vector where the first value represents patients with an age below 25 and the last value represents patients with an age of 90 or above. Values in between represent 5-year intervals.

We trained the model similarly to how we performed our ablation experiments. That is, we used the hyperparameters that gave the best performance on the validation set to train 11 differently seeded versions of the model and then report mean metrics and associated CIs on the MH-1813 test data. The results are as shown in table 4 below.

Supplementary Table 4. Overall performance on MH-1813 test data for the model that also takes patient age and sex as direct inputs. We also list the original performance of call-takers and the model (w/o sex and age) from the main manuscript for ease of comparison [mean (95% CI)]. NPV: negative predictive value, PPV: positive predictive value, FOR: false omission rate, CI: confidence interval.

	F1-score [%] ↑	Sensitivity [%] ↑	PPV [%] ↑	FOR [%] ↓ (1 - NPV)	FPR [%] ↓ (1 - specificity)
Call-takers	25.8 (23.7–27.9)	52.7 (49.2–56.4)	17.1 (15.5–18.6)	0.105 (0.094–0.116)	0.565 (0.539–0.590)
Model w/o sex and age	35.7 (35.0–36.4)	63.0 (62.0–64.1)	24.9 (24.3–25.5)	0.082 (0.079–0.085)	0.419 (0.413–0.426)
Model w/ sex and age	35.8 (35.1–36.5)	64.1 (63.1–65.1)	24.9 (24.3–25.4)	0.080 (0.077–0.082)	0.427 (0.421–0.434)

We note that the performance difference between the original model and the models with age and sex inputs is statistically insignificant ($p > 0.05$, paired approximate permutation test). We hypothesize this might be because information regarding the age and sex of the patient is, in many cases, already present in the transcript. Another reason might be that the patient's age and sex are less useful discriminators than other indicative factors. This latter hypothesis is supported by our occlusion analysis and Table 3 where no highly ranked words directly refer to the patient's age or sex.

It is important to note that in practice the information about patient sex and age is extracted from the patient's CPR number which is typically entered by the patient themselves while queuing for MH-1813. However, at call lines in countries without similar systems in place, such information may not be readily available and cannot be incorporated as an explicit input feature. The same is the case for the 1-1-2 call line in Denmark.

SUPPLEMENTARY RESULTS: MODEL WITHOUT MH-1813 TRAINING DATA

The ablation study in the main manuscript that examines the importance of the two different source domains (1-1-2 and MH-1813) includes only two of the three possible combinations of data sources, specifically: training on both 1-1-2 *and* MH-1813 data, and training *only* on MH-1813 *excluding* 1-1-2 data. For this reason, we have conducted an experiment that includes the remaining combination: training *only* on 1-1-2 *excluding* MH-1813 data.

Since the manuscript focuses on the MH-1813 line, the main purpose of experimenting with including and excluding the 1-1-2 data was to examine whether using the out-of-domain 1-1-2 data could improve stroke recognition performance of the machine learning framework on the MH-1813 data. This has interest since many prehospital call centres operate both a high acuity emergency line (like 1-1-2) and a low-acuity medical helpline (like MH-1813) which makes high-acuity data available for modelling and a potentially valuable data source. The ablation experiment performed here, on the other hand, has a different purpose. It aims to show whether a model to assist the MH-1813 helpline could be developed also in the hypothetical case of only having access to out-of-domain, high-acuity training data from 1-1-2. This prospect may be interesting for some call centres that, for instance, have only recently started operating a medical helpline, and so, do not have in-domain training data available.

We trained the models and provided the results of this experiment in table 5 below using the same methods as in the main manuscript. We tested the significance of these results using the same statistical significance testing methods as used and described in the main manuscript.

We see that the performance of the ensemble model trained only with 1-1-2 data compared to training only with MH-1813 data was worse in terms of sensitivity and FOR ($p < 0.0001$), but on-par in terms of F1-score and PPV, and better in terms of FPR ($p < 0.0001$). Compared to training with all data, training only on 1-1-2 was worse on all metrics ($p < 0.0001$) except FPR, where it was better ($p < 0.0001$). We note that the model still performed better than the call-takers in terms of F1-score, PPV, and FPR ($p < 0.0001$) and was on-par in terms of sensitivity and FOR. All tests for this comparison were paired approximate permutation tests.

In summary, training on only 1-1-2 data was only somewhat worse than training on only MH-1813 data, and still outperformed call-takers to some degree. This indicates that the domain shift between different call lines, even with different acuity levels, is small enough that naive domain transfer of models works well.

Supplementary Table 5. Overall performance on MH-1813 test data, performance without 1-1-2 training data, and performance without 1813 training data [mean (95% CI)]. NPV: negative predictive value, PPV: positive predictive value, FOR: false omission rate, CI: confidence interval.

	F1-score [%] ↑	Sensitivity [%] ↑	PPV [%] ↑	FOR [%] ↓ (1 - NPV)	FPR [%] ↓ (1 - specificity)
Overall					
Call-takers	25.8 (23.7–27.9)	52,7 (49,2–56.4)	17.1 (15.5–18.6)	0.105 (0.094–0.116)	0.565 (0.539–0.590)
Model	35.7 (35.0–36.4)	63.0 (62.0–64.1)	24.9 (24.3–25.5)	0.082 (0.079–0.085)	0.419 (0.413–0.426)
Without 1-1-2 training data (only MH-1813 data)					
Model	32.4 (31.8–33.1)	60.4 (59.3–61.4)	22.2 (21.6–22.7)	0.088 (0.085–0.091)	0.467 (0.460–0.474)
Without 1813 training data (only 1-1-2 data)					
Model	31.4 (30.7–32.1)	50.4 (49.3–51.4)	22.8 (22.2–23.4)	0.110 (0.106–0.113)	0.375 (0.369–0.381)

SUPPLEMENTARY RESULTS: DETAILED MODEL EXPLAINABILITY TABLES

In tables 6 and 7 we provide detailed versions of the data presented in the model explainability section in the main manuscript. Specifically, we include the Danish word, its English translation, the rank and impact scores and the number of occurrences of each word.

Supplementary Table 6. Mean impact for words with the largest positive rank score in calls predicted as stroke. Stroke predictions, D = 1,897.

	Word, w (Danish)	Translation (English)	Rank, $r^{(w)}$	Occurrences, $D^{(w)}$	Impact, $i^{(d, w)}$ Mean ± std.
1.	Ambulance	Ambulance	1.000	1,680	0.52 ± 0.51
2.	Blodprop	Blood clot	0.599	895	0.51 ± 0.58
3.	Venstre	Left	0.381	1,108	0.38 ± 0.4
4.	Højre	Right	0.326	1,050	0.31 ± 0.42
5.	Dobbeltsyn	Double vision	0.247	84	1.01 ± 1.26
6.	Ordene	The words	0.217	344	0.6 ± 0.45
7.	Pludselig	Suddenly	0.142	783	0.29 ± 0.28
8.	Arm	Arm	0.140	709	0.3 ± 0.3
9.	Side	Side	0.125	1,139	0.23 ± 0.21
10.	Apopleksi	Stroke	0.102	117	0.33 ± 0.82
11.	Dobbelt	Double	0.102	113	0.54 ± 0.72
12.	Styre	Control	0.092	134	0.63 ± 0.46
13.	Opkald	Call	0.067	39	0.18 ± 1.22
14.	Følelsesløs	Numb	0.065	94	0.53 ± 0.58
15.	Minutter	Minutes	0.064	763	0.22 ± 0.16
16.	Talebesvær	Difficulties speaking	0.063	44	0.87 ± 0.72
17.	Hjerneblødning	Haemorrhagic stroke	0.060	133	0.4 ± 0.49
18.	Hånd	Hand	0.057	297	0.28 ± 0.31
19.	Ambulancen	The ambulance	0.055	521	0.21 ± 0.23
20.	Snøvler	Slurred speech	0.052	58	0.71 ± 0.54
21.	Blodpropper	Blood clots	0.051	224	0.27 ± 0.36
22.	Hurtigt	Fast	0.048	663	0.18 ± 0.18
23.	Udtrykke	Express	0.044	44	0.59 ± 0.74
24.	Blodfortyndende	Blood thinner	0.044	259	0.32 ± 0.22
25.	Usammenhængende	Incoherent	0.043	15	1.14 ± 1.13
26.	Skæv	Lopsided	0.039	211	0.29 ± 0.28
27.	Nedsat	Reduced	0.038	528	0.14 ± 0.21
28.	Hænger	Hangs	0.036	628	0.15 ± 0.17
29.	Forbigående	Transient	0.035	48	0.52 ± 0.62
30.	Vrøvler	Not making sense	0.033	14	1.13 ± 0.89

Supplementary Table 7. Mean impact for words with the largest negative rank score in calls predicted as non-stroke. Non-stroke predictions, D = 342,133

	Word, *w* (Danish)	Translation (English)	Rank, $r^{(w)}$	Occurrences, $D^{(w)}$	Impact, $i^{(d, w)}$ mean ± std.
1.	Stivkrampe	Tetanus	-1.000	4378	-19.40 ± 10.61
2.	Gravid	Pregnant	-0.901	8749	-12.08 ± 8.64
3.	Skåret	Cut	-0.772	7592	-11.98 ± 8.61
4.	Forbinding	Bandage	-0.569	4561	-12.87 ± 10.08
5.	Amager	Amager (a location)	-0.566	23776	-5.60 ± 4.43
6.	Klokken	O'clock	-0.535	94436	-2.22 ± 2.69
7.	Skadestuen	The emergency room	-0.486	42809	-3.72 ± 3.23
8.	Politiet	The police	-0.413	2903	-10.73 ± 13.77
9.	Hævet	Swollen	-0.388	60559	-2.84 ± 2.38
10.	Håndkøb	over the counter (otc)	-0.372	4641	-11.64 ± 6.00
11.	Halsen	The neck	-0.366	30151	-3.33 ± 3.86
12.	Feber	Fever	-0.361	112586	-1.94 ± 1.76
13.	Recept	Prescription	-0.334	5450	-9.87 ± 5.82
14.	Centimetre	Centimetre	-0.311	12026	-6.01 ± 4.39
15.	Knæet	The knee	-0.300	8875	-6.12 ± 5.91
16.	Apoteket	The pharmacy	-0.267	10085	-6.05 ± 4.49
17.	Maven	The stomach	-0.267	42105	-2.36 ± 2.82
18.	Psykiatrisk	Psychiatric	-0.263	3688	-8.99 ± 8.49
19.	Lungebetændelse	Pneumonia	-0.231	7597	-5.79 ± 5.62
20.	Mavesmerter	Stomach pain	-0.209	10551	-5.12 ± 4.02
21.	Afføring	Stool	-0.199	19155	-3.40 ± 3.27
22.	Ribbenene	The ribs	-0.195	3928	-8.26 ± 6.18
23.	Bløde	Bleed	-0.194	10501	-4.88 ± 3.97
24.	Bløder	Bleeding	-0.193	24313	-2.90 ± 2.93
25.	Ribben	Ribs	-0.189	2941	-8.96 ± 7.56
26.	Brækket	Broken	-0.183	19415	-3.49 ± 2.83
27.	Betændelse	Inflammation	-0.181	10050	-5.27 ± 3.30
28.	Forkølet	Common cold	-0.161	8127	-5.31 ± 3.75
29.	Morgen	Morning *or* morrow	-0.160	78558	-1.23 ± 1.70
30.	Hævelse	Swelling	-0.159	17762	-3.71 ± 2.32

SUPPLEMENTARY RESULTS: FINE-TUNING OF DANISH BERT MODEL FOR STROKE RECOGNITION

During the preliminary experimental phase, we fine-tuned a BERT model pre-trained on Danish text from CommonCrawl, Wikipedia, OpenSubtitles, and other Danish online forums (available at https://github.com/certainlyio/nordic_bert). The model was fine-tuned for 10,000 updates using linear learning rate warm-up (1,000 updates) and decay (9,000 updates). The maximum learning rate was set to 5×10^{-5} and an accumulated batch size of 128. The maximum sequence length of the pre-trained model was 512 input tokens. To accommodate longer input sequences, which was necessary for our dataset, we concatenated several copies of the original positional embedding matrix. The results of the final model are presented in table 8.

We see that the fine-tuned BERT model performs slightly worse across F1-score, sensitivity, PPV and FOR ($p < 0.0001$, paired approximate permutation test), but better in terms of FPR ($p < 0.0001$, paired approximate permutation test), compared the MLP model presented in the main manuscript. As described in the discussion section, we hypothesize that the number of stroke positives was too small for these advanced models to learn more complex patterns than the MLP ensemble. In addition, the BERT model would likely benefit from pre-training on text data from the target domain, or a domain close to it, rather than various online fora.

Supplementary Table 8. Overall performance on MH-1813 test data for the fine-tuned BERT model described in the revised discussion of the paper. Includes performance of the call-takers and the multi-layer perceptron (MLP) from the main manuscript for ease of comparison [mean (95% CI)]. NPV: negative predictive value, PPV: positive predictive value, FOR: false omission rate, CI: confidence interval.

	F1-score [%] ↑	Sensitivity [%] ↑	PPV [%] ↑	FOR [%] ↓ (1 - NPV)	FPR [%] ↓ (1 - specificity)
Call-takers	25.8 (23.7–27.9)	52.7 (49.2–56.4)	17.1 (15.5–18.6)	0.105 (0.094–0.116)	0.565 (0.539–0.590)
MLP	35.8 (35.1–36.5)	64.1 (63.1–65.1)	24.9 (24.3–25.4)	0.080 (0.077–0.082)	0.427 (0.421–0.434)
BERT (fine-tuned)	33.8 (31.5–36.2)	57.5 (53.9–60.9)	23.9 (21.9–25.9)	0.094 (0.084–0.104)	0.403 (0.381–0.424)

SUPPLEMENTARY RESULTS: SIMULATION OF A PROSPECTIVE STUDY ON 2021 DATA

Method

The machine learning frameworks can be deployed in different forms in clinical practice. To assess the potential outcomes of deploying this framework in a future prospective study, we performed an experiment using the 2021 test data to simulate different scenarios. Each scenario included two main variables.

I. Presentation of the model prediction to the call taker.
II. How does prediction influence the diagnostic code the call taker assigns to the call?

There are two primary options as to when the model prediction is presented (I):

1. Notify the call taker of potential false positive or negative stroke cases after call end.
2. Notify the call taker of potential false positive or negative stroke cases during the call.

Option 1 is identical to the method used in the main study. In option 2, predictions are made during the call based only on partial transcriptions. We implemented option 2 in such a manner that the model predicted every time 50 new words were transcribed and added to the transcript. A stroke positive was triggered only when three consecutive positive predictions were made (i.e., without intermediate negative stroke predictions). In other words, the sigmoid activation of the model had to remain above 0.5 for three consecutive predictions, for example, after 150, 200, and 250 words were transcribed.

As we can only assume how call takers are influenced by model predictions (II), precisely evaluating the hypothetical performance of call takers when supported by a machine learning framework is impossible. Furthermore, option 2 may influence the conversation, further complicating matters. Therefore, we report the results combining the call taker and the model under the following two assumptions:

A. Call takers change any stroke prediction from negative to positive if the model predicts a positive (call takers mirror model positives).
B. Call takers change any stroke prediction from positive to negative if the model predicts a negative (call takers mirror model negatives).

By definition, method A tended to increase sensitivity and decrease PPV, whereas method B tended to decrease sensitivity and increase PPV.

We also report the results of the model itself (C). This method corresponds to call takers mirroring the model predictions exactly. This is not feasible in practice, although technically possible, because the conversation and instructions given to patients may conflict with the actions taken by the call taker after hanging up. Method 1.C is identical to the method employed in the main text, and we have copied the same results here for easier comparison.

Results

As expected (table 9), method 2.C (raw model predictions during calls) yielded slightly worse results than 1.C (raw model predictions after calls). Compared with method C, method A (call

takers mirror model positives) led to increased sensitivity and decreased PPV, whereas method B (call takers mirror model negatives) led to decreased sensitivity and increased PPV, as expected. The numerical changes compared with method C are quite large because, in our simulation, the call taker is assumed to strictly follow methods A or B without divergence.

Supplementary Table 9. Overall performance of model, call takers and simulated combinations of model and call takers on MH-1813 test data. C: call taker, M: model, C+M: call taker supported by the model (simulated). NPV: negative predictive value, PPV: positive predictive value, FOR: false omission rate, CI: confidence interval.

Predictor	C	M	M	C+M	C+M	C+M	C+M
When	–	After call	During call	After call	After call	During call	During call
Method	–	1.C	2.C	1.A	1.B	2.A	2.B
F1-score [%] ↑	25.8<br>(23.7–27.9)	35.7<br>(35.0–36.4)	33.1<br>(32.4–33.7)	28.9<br>(28.3–29.5)	33.3<br>(32.5–34.1)	27.6<br>(27.0–28.1)	32.7<br>(31.8–33.5)
Sensitivity [%] ↑	52.7<br>(49.2–56.4)	63.0<br>(62.0–64.1)	58.7<br>(57.7–59.8)	72.4<br>(71.5–73.3)	43.4<br>(42.3–44.5)	72.3<br>(71.4–73.3)	39.1<br>(38.1–40.1)
PPV [%] ↑	17.1<br>(15.5–18.6)	24.9<br>(24.3–25.5)	23.0<br>(22.5–23.6)	18.0<br>(17.6–18.4)	27.0<br>(26.3–27.8)	17.0<br>(16.7–17.4)	28.1<br>(27.3–28.9)
FOR [%] ↓<br>(1 – specificity)	0.105<br>(0.094–0.116)	0.082<br>(0.079–0.085)	0.091<br>(0.088–0.094)	0.061<br>(0.059–0.064)	0.125<br>(0.121–0.129)	0.061<br>(0.059–0.064)	0.134<br>(0.131–0.138)
FPR [%] ↓<br>(1 – NPV)	0.565<br>(0.539–0.590)	0.419<br>(0.413–0.426)	0.432<br>(0.426–0.439)	0.726<br>(0.717–0.735)	0.258<br>(0.253–0.263)	0.776<br>(0.767–0.786)	0.221<br>(0.216–0.226)

Method 1.A (call takers mirror model positives after a call) yields a better F1-score, sensitivity, PPV, and FOR than call takers alone, although at the cost of a slightly higher FPR. This stands in contrast to methods 1.B, 2.A, and 2.B where either the sensitivity or the PPV is worse for the combined system than for call takers alone. Regardless, the F1-score (harmonic mean of sensitivity and PPV) is higher for all methods of combining call takers and model (1.A through 2.C).

These findings highlight that the implementation strategy selected for practice can substantially affect performance. Therefore, it may be possible to implement the system in a way that improves stroke recognition in practice.

SUPPLEMENTARY DISCUSSION: RESEARCH IN CONTEXT

Evidence before this study

We searched the PubMed database for articles published in any language up to 9 May 2023 using the following terms: *stroke* AND (*artificial intelligence* OR *machine learning* OR *deep learning*) AND (*EMS* OR *emergency medical services* OR *dispatch* OR *telephone*). We identified 88 articles, none of which reported the results of machine learning-based stroke recognition during telephone calls.

One study assessed the potential impact of using speech classification software for stroke recognition by extrapolating results from a similar solution for recognising cardiac arrest. Several authors of this study co-authored the article; however, it did not report the results of an actual novel machine learning framework. The remaining articles primarily reported the use of machine learning in imaging diagnostics, stroke recognition using movement-tracking mobile devices, and the development of stroke recognition tools using other non-audio data.

Added value of this study

This study is the first to investigate the use of a machine learning framework for stroke recognition in medical helpline calls. The study's results can be replicated in other call lines and for other acute illnesses. A machine learning framework has been previously described for out-of-hospital cardiac arrest; however, our results illustrate the feasibility of employing machine learning for detecting stroke – a more complex acute condition.

Implications of all the available evidence

Implementing the framework described in this study could lead to improved recognition of patients with stroke during initial contact with health services. Improving recognition would result in more patients being eligible for advanced stroke treatment and better overall outcomes owing to faster referral to a stroke unit.

SUPPLEMENTARY DISCUSSION: RESEARCH IN CONTEXT SEARCH TERM RESULTS

The search terms *stroke* AND (*artificial intelligence* OR *machine learning* OR *deep learning*) AND (*EMS* OR *emergency medical services* OR *dispatch* OR *telephone*) yielded 88 articles in PubMed. These articles are listed below:

1. Soun JE, Chow DS, Nagamine M et al. Artificial intelligence and acute stroke imaging. *AJNR. American journal of neuroradiology* 2021; **42**: 2–11.
2. Murray NM, Unberath M, Hager GD, Hui FK. Artificial intelligence to diagnose ischemic stroke and identify large vessel occlusions: a systematic review. *Journal of neurointerventional surgery* 2020; **12**: 156–64.
3. Shlobin NA, Baig AA, Waqas M et al. Artificial intelligence for large-vessel occlusion stroke: a systematic review. *World neurosurgery* 2022; **159**: 207–20.e1.
4. Linder SM, Rosenfeldt AB, Bay RC, Sahu K, Wolf SL, Alberts JL. Improving quality of life and depression after stroke through telerehabilitation. *The American journal of occupational therapy: official publication of the American Occupational Therapy Association* 2015; **69**: 6902290020p1-10.
5. Humphreys K, Shover CL, Andrews CM et al. Responding to the opioid crisis in North America and beyond: recommendations of the Stanford-Lancet Commission. *Lancet (London, England)* 2022; **399**: 555–604.
6. Fassbender K, Lesmeister M, Merzou F. Prehospital stroke management and mobile stroke units. *Current opinion in neurology* 2023; **36**: 140–6.
7. McDonough RV, Ospel JM, Majoie CBLM et al. Clinical outcome of patients with mild pre-stroke morbidity following endovascular treatment: a HERMES substudy. *Journal of neurointerventional surgery* 2023; **15**: 214–20.
8. Bat-Erdene BO, Saver JL. Automatic acute stroke symptom detection and emergency medical systems alerting by mobile health technologies: a review. *Journal of stroke and cerebrovascular diseases: the official journal of National Stroke Association* 2021; **30**: 105826.
9. Bonini N, Vitolo M, Imberti JF et al. Mobile health technology in atrial fibrillation. *Expert review of medical devices* 2022; **19**: 327–40.
10. Scholz ML, Collatz-Christensen H, Blomberg SNF, Boebel S, Verhoeven J, Krafft T. Artificial intelligence in Emergency Medical Services dispatching: assessing the potential impact of an automatic speech recognition software on stroke detection taking the Capital Region of Denmark as case in point. *Scandinavian journal of trauma, resuscitation and emergency medicine* 2022; **30**: 36.
11. Gupta R, Krishnam SP, Schaefer PW, Lev MH, Gonzalez RG. An East Coast perspective on artificial intelligence and machine learning: part 2: ischemic stroke imaging and triage. *Neuroimaging clinics of North America* 2020; **30**: 467–78.
12. Esbjörnsson M, Ullberg T. Safety and usability of wearable accelerometers for stroke detection the STROKE ALARM PRO 1 study. *Journal of stroke and cerebrovascular diseases: the official journal of National Stroke Association* 2022; **31**: 106762.

13. Fast L, Temuulen U, Villringer K et al. Machine learning-based prediction of clinical outcomes after first-ever ischemic stroke. *Frontiers in neurology* 2023; **14**: 1114360.
14. Gupta R, Krishnam SP, Schaefer PW, Lev MH, Gilberto Gonzalez R. An East Coast perspective on artificial intelligence and machine learning: part 1: hemorrhagic stroke imaging and triage. *Neuroimaging clinics of North America* 2020; **30**: 459–66.
15. Cai T, Ni H, Yu M et al. DeepStroke: An efficient stroke screening framework for emergency rooms with multimodal adversarial deep learning. *Medical image analysis* 2022; **80**: 102522.
16. Zachrison KS, Dhand A, Schwamm LH, Onnela JP. A network approach to stroke systems of care. *Circulation. Cardiovascular quality and outcomes* 2019; **12**: e005526.
17. O'Connell GC, Walsh KB, Smothers CG et al. Use of deep artificial neural networks to identify stroke during triage via subtle changes in circulating cell counts. *BMC neurology* 2022; **22**: 206.
18. Yang L, Liu Q, Zhao Q, Zhu X, Wang L. Machine learning is a valid method for predicting prehospital delay after acute ischemic stroke. *Brain and behavior* 2020; **10**: e01794.
19. Aldridge CM, McDonald MM, Wruble M et al. Human vs. machine learning based detection of facial weakness using video analysis. *Frontiers in neurology* 2022; **13**: 878282.
20. Lachance CC, Ford C. Portable stroke detection devices for patients with stroke symptoms: a review of diagnostic accuracy and cost-effectiveness. *Canadian Agency for Drugs and Technologies in Health* 2019
21. Fardoun HM, Mashat AS. Methodologies, models and algorithms for patients rehabilitation. *Methods of information in medicine* 2016; **55**: 60–4.
22. Wibring K, Magnusson C, Axelsson C, Lundgren P, Herlitz J, Andersson Hagiwara M. Towards definitions of time-sensitive conditions in prehospital care. *Scandinavian journal of trauma, resuscitation and emergency medicine* 2020; **28**: 7.
23. Mayampurath A, Parnianpour Z, Richards CT et al. Improving prehospital stroke diagnosis using natural language processing of paramedic reports. *Stroke* 2021; **52**: 2676–9.
24. Morey JR, Zhang X, Yaeger KA et al. Real-world experience with artificial intelligence-based triage in transferred large vessel occlusion stroke patients. *Cerebrovascular diseases (Basel, Switzerland)* 2021; **50**: 450–5.
25. Sung SF, Hung LC, Hu YH. Developing a stroke alert trigger for clinical decision support at emergency triage using machine learning. *International journal of medical informatics* 2021; **152**: 104505.
26. Zhang Z, Zhou D, Zhang J et al. Multilayer perceptron-based prediction of stroke mimics in prehospital triage. *Scientific reports* 2022; **12**: 17994.
27. Park E, Lee K, Han T, Nam HS. Automatic grading of stroke symptoms for rapid assessment using optimized machine learning and 4-limb kinematics: clinical validation study. *Journal of medical Internet research* 2020; **22**: e20641.
28. Chen M, Tan X, Padman R. A machine learning approach to support urgent stroke triage using administrative data and social determinants of health at hospital presentation: retrospective study. *Journal of medical Internet research* 2023; **25**: e36477.

29. Uchida K, Kouno J, Yoshimura S et al. Development of machine learning models to predict probabilities and types of stroke at prehospital stage: the Japan urgent stroke triage score using machine learning (JUST-ML). *Translational stroke research* 2022; **13**: 370–81.
30. Finck T, Schinz D, Grundl L et al. Automated detection of ischemic stroke and subsequent patient triage in routinely acquired head CT. *Clinical neuroradiology* 2022; **32**: 419-26.
31. Sangal RB, Fodeh S, Taylor A et al. Identification of patients with nontraumatic intracranial hemorrhage using administrative claims data. *Journal of stroke and cerebrovascular diseases: the official journal of National Stroke Association* 2020; **29**: 105306.
32. Adedinsewo D, Carter RE, Attia Z et al. Artificial intelligence-enabled ECG algorithm to identify patients with left ventricular systolic dysfunction presenting to the emergency department with dyspnea. *Circulation. Arrhythmia and electrophysiology* 2020; **13**: e008437.
33. Liu L, Yu B, Han M, Yuan S, Wang N. Mild cognitive impairment understanding: an empirical study by data-driven approach. *BMC bioinformatics* 2019; **20**: 481.
34. Hayashi Y, Shimada T, Hattori N et al. A prehospital diagnostic algorithm for strokes using machine learning: a prospective observational study. *Scientific reports* 2021; **11**: 20519.
35. Grant L, Joo P, Nemnom MJ, Thiruganasambandamoorthy V. Machine learning versus traditional methods for the development of risk stratification scores: a case study using original Canadian Syncope Risk Score data. *Internal and emergency medicine* 2022; **17**: 1145–53.
36. van Meenen LCC, van Stigt MN, Marquering HA et al. Detection of large vessel occlusion stroke with electroencephalography in the emergency room: first results of the ELECTRA-STROKE study. *Journal of neurology* 2022; **269**: 2030–8.
37. van Meenen LCC, den Hartog SJ, Groot AE et al. Relationship between primary stroke center volume and time to endovascular thrombectomy in acute ischemic stroke. *European journal of neurology* 2021; **28**: 4031–8.
38. Chien HC, Yang TL, Juang WC, Chen YA, Li YJ, Chen CY. Pilot report for intracranial hemorrhage detection with deep learning implanted head computed tomography images at emergency department. *Journal of medical systems* 2022; **46**: 49.
39. Huo D, Leppert M, Pollard R et al. Large vessel occlusion prediction in the emergency department with national institutes of health stroke scale components: a machine learning approach. *Journal of stroke and cerebrovascular diseases: the official journal of National Stroke Association* 2021; **30**: 106030.
40. Wu MR, Chen YT, Li ZX et al. Dysphagia screening and pneumonia after subarachnoid hemorrhage: findings from the Chinese stroke center alliance. *CNS neuroscience & therapeutics* 2022; **28**: 913–21.
41. Bacchi S, Oakden-Rayner L, Zerner T, Kleinig T, Patel S, Jannes J. Deep learning natural language processing successfully predicts the cerebrovascular cause of transient ischemic attack-like presentations. *Stroke* 2019; **50**: 758–60.
42. Heldt FS, Vizcaychipi MP, Peacock S et al. Early risk assessment for COVID-19 patients from emergency department data using machine learning. *Scientific reports* 2021; **11**: 4200.

43. Lu Z, Xiong Y, Yang K et al. What predicts large vessel occlusion in mild stroke patients. *BMC neurology* 2023; **23**: 29.
44. Marijon E, Garcia R, Narayanan K, Karam N, Jouven X. Fighting against sudden cardiac death: need for a paradigm shift-adding near-term prevention and pre-emptive action to long-term prevention. *European heart journal* 2022; **43**: 1457–64.
45. You J, Yu PLH, Tsang ACO et al. 3D dissimilar-Siamese-u-net for hyperdense Middle cerebral artery sign segmentation. *Computerized medical imaging and graphics: the official journal of the Computerized Medical Imaging Society* 2021; **90**: 101898.
46. Moral-Munoz JA, Zhang W, Cobo MJ, Herrera-Viedma E, Kaber DB. Smartphone-based systems for physical rehabilitation applications: a systematic review. *Assistive technology: the official journal of RESNA* 2021; **33**: 223–36.
47. Salman OH, Aal-Nouman MI, Taha ZK, Alsabah MQ, Hussein YS, Abdelkareem ZA. Formulating multi diseases dataset for identifying, triaging and prioritizing patients to multi medical emergency levels: Simulated dataset accompanied with codes. *Data in brief* 2021; **34**: 106576.
48. Desai A, Zumbo A, Giordano M et al. Word2vec Word embedding-based artificial intelligence model in the triage of patients with suspected diagnosis of major ischemic stroke: a feasibility study. *International journal of environmental research and public health* 2022; **19**: 15295.
49. Rinkel LA, Prick JCM, Slot RER et al. Impact of the COVID-19 outbreak on acute stroke care. *Journal of neurology* 2021; **268**: 403–8.
50. Chilamkurthy S, Ghosh R, Tanamala S et al. Deep learning algorithms for detection of critical findings in head CT scans: a retrospective study. *Lancet (London, England)* 2018; **392**: 2388–96.
51. Kramer NM, Demaerschalk BM. A novel application of teleneurology: robotic telepresence in supervision of neurology trainees. *Telemedicine journal and e-health: the official journal of the American Telemedicine Association* 2014; **20**: 1087–92.
52. Yadav K, Sarioglu E, Choi HA, Cartwright WB, Hinds PS, Chamberlain JM. Automated outcome classification of computed tomography imaging reports for pediatric traumatic brain injury. *Academic emergency medicine: official journal of the Society for Academic Emergency Medicine* 2016; **23**: 171–8.
53. van Meenen LCC, Riedijk F, Stolp J et al. Pre- and interhospital workflow times for patients with large vessel occlusion stroke transferred for endovasvular thrombectomy. *Frontiers in neurology* 2021; **12**: 730250.
54. Saberi-Movahed F, Mohammadifard M, Mehrpooya A et al. Decoding clinical biomarker space of COVID-19: Exploring matrix factorization-based feature selection methods. *Computers in biology and medicine* 2022; **146**: 105426.
55. Shimada T, Matsubara K, Koyama D et al. Development of evaluation system for cerebral artery occlusion in emergency medical services: noninvasive measurement and utilization of pulse waves. *Scientific reports* 2023; **13**: 3339.

56. Gil-Jardiné C, Chenais G, Pradeau C et al. Trends in reasons for emergency calls during the COVID-19 crisis in the department of Gironde, France using artificial neural network for natural language classification. *Scandinavian journal of trauma, resuscitation and emergency medicine* 2021; **29**: 55.
57. Santala OE, Lipponen JA, Jäntti H et al. Continuous mHealth Patch Monitoring for the Algorithm-Based Detection of Atrial Fibrillation: Feasibility and Diagnostic Accuracy Study. *JMIR cardio* 2022; **6**: e31230.
58. O'Brien MK, Shawen N, Mummidisetty CK et al. Activity recognition for persons with stroke using mobile phone technology: toward improved performance in a home setting. *Journal of medical Internet research* 2017; **19**: e184.
59. Bateman RM, Sharpe MD, Jagger JE et al. 36th international symposium on intensive care and emergency medicine: Brussels, Belgium. 15-18 March 2016. *Critical care (London, England)* 2016; **20**: 94.
60. Modica CM, Johnson BR, Zalewski C et al. Hearing loss and irritability reporting without vestibular differences in explosive breaching professionals. *Frontiers in neurology* 2020; **11**: 588377.
61. Palmcrantz S, Plantin J, Borg J. Factors affecting the usability of an assistive soft robotic glove after stroke or multiple sclerosis. *Journal of rehabilitation medicine* 2020; **52**: jrm00027.
62. Mitsopoulos K, Fiska V, Tagaras K et al. NeuroSuitUp: System architecture and validation of a motor rehabilitation wearable robotics and serious game platform. *Sensors (Basel, Switzerland)* 2023; **23**: 3281.
63. Meng G, Tan Y, Fang M, Yang H, Liu X, Zhao Y. Meteorological factors related to emergency admission of elderly stroke patients in shanghai: analysis with a multilayer perceptron neural network. *Medical science monitor: international medical journal of experimental and clinical research* 2015; **21**: 3600–7.
64. Iglesias JE, Schleicher R, Laguna S et al. Quantitative Brain Morphometry of Portable Low-Field-Strength MRI Using Super-Resolution Machine Learning. *Radiology* 2023; **306**: e220522.
65. Lai F. Stroke networks based on robotic telepresence. *Journal of telemedicine and telecare* 2009; **15**: 135–6
66. Kim D, Oh J, Im H, Yoon M, Park J, Lee J. Automatic classification of the Korean triage acuity scale in simulated emergency rooms using speech recognition and natural language processing: a proof of concept study. *Journal of Korean medical science* 2021; **36**: e175.
67. Jonsson A, Binongo J, Patel P et al. Mastering the learning curve for robotic-assisted coronary artery bypass surgery. *The Annals of thoracic surgery* 2023; **115**: 1118–25.
68. Knowlton SM, Sencan I, Aytar Y et al. Sickle cell detection using a smartphone. *Scientific reports* 2015; **5**: 15022.
69. Che F, Liu Y, Gong X et al. Extracranial carotid plaque hemorrhage is independently associated with poor 3-month functional outcome after acute ischemic stroke-a prospective cohort study. *Frontiers in neurology* 2021; **12**: 780436.

70. Boers AMM, Jansen IGH, Brown S et al. Mediation of the relationship between endovascular therapy and functional outcome by follow-up infarct volume in patients with acute ischemic stroke. *JAMA neurology* 2019; **76**: 194–202.
71. Stone JR, Avants BB, Tustison NJ et al. Functional and structural neuroimaging correlates of repetitive low-level blast exposure in career breachers. *Journal of neurotrauma* 2020; **37**: 2468–81.
72. Rickards CA, Vyas N, Ryan KL et al. Are you bleeding? Validation of a machine-learning algorithm for determination of blood volume status: application to remote triage. *Journal of applied physiology (Bethesda, Md. : 1985)* 2014; **116**: 486–94.
73. Edwards KA, Greer K, Leete J et al. Neuronally-derived tau is increased in experienced breachers and is associated with neurobehavioral symptoms. *Scientific reports* 2021; **11**: 19527.
74. Cheng AL, Liu J, Bravo S, Miller JC, Pahlevan NM. Screening left ventricular systolic dysfunction in children using intrinsic frequencies of carotid pressure waveforms measured by a novel smartphone-based device. *Physiological measurement* 2023; **44**.
75. Grunwald IQ, Kulikovski J, Reith W et al. Collateral automation for triage in stroke: evaluating automated scoring of collaterals in acute stroke on computed tomography scans. *Cerebrovascular diseases (Basel, Switzerland)* 2019; **47**: 217–22.
76. Hyakutake K, Morishita T, Saita K et al. Effects of home-based robotic therapy involving the single-joint hybrid assistive limb robotic suit in the chronic phase of stroke: a pilot study. *BioMed research international* 2019; **2019**: 5462694.
77. Murray C, Ortiz E, Kubin C. Application of a robot for critical care rounding in small rural hospitals. *Critical care nursing clinics of North America* 2014; **26**: 477–85.
78. Edwards KA, Leete JJ, Tschiffely AE et al. Blast exposure results in tau and neurofilament light chain changes in peripheral blood. *Brain injury* 2020; **34**: 1213–21.
79. Oresko JJ, Duschl H, Cheng AC. A wearable smartphone-based platform for real-time cardiovascular disease detection via electrocardiogram processing. *IEEE transactions on information technology in biomedicine : a publication of the IEEE Engineering in Medicine and Biology Society* 2010; **14**: 734–40.
80. Alcoceba-Herrero I, Coco-Martín MB, Leal-Vega L et al. Randomized controlled trial evaluating the benefit of a novel clinical decision support system for the management of COVID-19 patients in home quarantine: a study protocol. *International journal of environmental research and public health* 2023; **20**: 2300.
81. van Meenen LCC, Groot AE, Venema E et al. Interhospital transfer vs. direct presentation of patients with a large vessel occlusion not eligible for IV thrombolysis. *Journal of neurology* 2020; **267**: 2142–50.
82. Forghani R, Gupta R. Case of the Season: Artificial intelligence in clinical practice-large vessel occlusion triage in stroke imaging. *Seminars in roentgenology* 2023; **58**: 147–51.
83. Jabbour P, Gonzalez LF, Tjoumakaris S, Randazzo C, Rosenwasser R. Stroke in the robotic era. *World neurosurgery* 2010; **73**: 603–4.
84. Larkin H. On the spot when doc is not. *Hospitals & health networks* 2008; **82**: 41–2.

85. Jiang Y, Guarino P, Ma S et al. Bayesian accrual prediction for interim review of clinical studies: open source R package and smartphone application. *Trials* 2016; **17**: 336.
86. Lee JH, Oh BJ, Ahn JY et al. Effectiveness of automatic acute stroke alert system based on UMLS mapped local terminology codes at emergency department. *AMIA ... Annual Symposium proceedings. AMIA Symposium* 2008; 1018.
87. Robot saves ED stroke patients, addresses subspecialist shortage. *ED management: the monthly update on emergency department management* 2011; **23**: 13–5.
88. Nagy Z, Simon P, Sipos E, Kozmann G. The main elements of the information system of the National Stroke Program (Smart Card - Telecommunication - Knowledge Bases). *Medinfo. MEDINFO* 1995; **8 Pt 2**: 1496–9.

REFERENCES

1. Dwass M. Modified Randomization Tests for Nonparametric Hypotheses. *Ann Math Stat* 1957; 28: 181–187.

2. Eden T, Yates F. On the validity of Fisher’s z test when applied to an actual example of non-normal data. (With five text-figures.). *J Agric Sci* 1933; 23: 6–17.
