# Supplementary material for: A retrospective study on machine learning-assisted stroke recognition for medical helpline calls
Source: NPJ Digit Med. 2023 Dec 19;6:235. doi: 10.1038/s41746-023-00980-y (PMC10730829; doi:10.1038/s41746-023-00980-y)
Supplement: Supplementary file 2 — Reporting Summary [file 41746_2023_980_MOESM2_ESM.pdf]

nature portfolio

Corresponding author(s): Helle Collatz Christensen

Last updated by author(s): Nov 15, 2023

Reporting Summary

Nature Portfolio wishes to improve the reproducibility of the work that we publish. This form provides structure for consistency and transparency in reporting. For further information on Nature Portfolio policies, see our Editorial Policies and the Editorial Policy Checklist.

Statistics

For all statistical analyses, confirm that the following items are present in the figure legend, table legend, main text, or Methods section.

n/a	Confirmed	
☐	☒	The exact sample size (*n*) for each experimental group/condition, given as a discrete number and unit of measurement
☐	☒	A statement on whether measurements were taken from distinct samples or whether the same sample was measured repeatedly
☐	☒	The statistical test(s) used AND whether they are one- or two-sided<br>*Only common tests should be described solely by name; describe more complex techniques in the Methods section.*
☐	☒	A description of all covariates tested
☐	☒	A description of any assumptions or corrections, such as tests of normality and adjustment for multiple comparisons
☐	☒	A full description of the statistical parameters including central tendency (e.g. means) or other basic estimates (e.g. regression coefficient) AND variation (e.g. standard deviation) or associated estimates of uncertainty (e.g. confidence intervals)
☐	☒	For null hypothesis testing, the test statistic (e.g. *F*, *t*, *r*) with confidence intervals, effect sizes, degrees of freedom and *P* value noted<br>*Give P values as exact values whenever suitable.*
☒	☐	For Bayesian analysis, information on the choice of priors and Markov chain Monte Carlo settings
☒	☐	For hierarchical and complex designs, identification of the appropriate level for tests and full reporting of outcomes
☒	☐	Estimates of effect sizes (e.g. Cohen's *d*, Pearson's *r*), indicating how they were calculated

Our web collection on statistics for biologists contains articles on many of the points above.

Software and code

Policy information about availability of computer code

Data collection	We used Python version 3.8.10 to access CEMS databases (Cosmos and Postgres) and Azure Datalakes and used the PyMongo package version 4.3.3 to execute data extraction commands in Azure Kubernetes.
Data analysis	We used Python version 3.8.10. To train the neural network models we used PyTorch version 1.12.1+cu113. To perform bag-of-words vectorization we used SciKit-Learn version 1.2.2. To perform data analysis, plotting and testing we used NumPy version 1.23.5, Pandas version 1.5.3, Matplotlib version 3.7.1 and SciPy version 1.10.1.

For manuscripts utilizing custom algorithms or software that are central to the research but not yet described in published literature, software must be made available to editors and reviewers. We strongly encourage code deposition in a community repository (e.g. GitHub). See the Nature Portfolio guidelines for submitting code & software for further information.

Data

Policy information about availability of data

All manuscripts must include a data availability statement. This statement should provide the following information, where applicable:

- Accession codes, unique identifiers, or web links for publicly available datasets
- A description of any restrictions on data availability
- For clinical datasets or third party data, please ensure that the statement adheres to our policy

The datasets used to evaluate call taker performance and to train and evaluate the machine learning framework are legally restricted by Danish patient privacy and

secrecy laws and are therefore not publicly available. The data can be made available from the date of publication but requires a Data Access Agreement, which is examined and approved by the ethics committees who approved this research. For the same reason, the machine learning framework trained in this study is not publicly available, but instructions on how to train it are included in the main manuscript and supplementary material. The source code can be shared using a Creative Commons NC-ND 4.0 international licence upon reasonable written request to the corresponding author and requires a data use agreement.

Research involving human participants, their data, or biological material

Policy information about studies with human participants or human data. See also policy information about sex, gender (identity/presentation), and sexual orientation and race, ethnicity and racism.

Reporting on sex and gender	We have complied with this, using sex for biological sex in the entire manuscript. Where appropriate, we have investigated differences in recognition between sexes.
Reporting on race, ethnicity, or other socially relevant groupings	We did not report on race or ethnicity in the manuscript. We did report on different age groups as these are highly relevant for stroke.
Population characteristics	We only used epidemiological registry data for this study. The available population characteristics are reported in table 1.
Recruitment	We only used epidemiological registry data for this study.
Ethics oversight	The study was approved by the Danish Data Protection Agency (P-2021-475). Approval from the Scientific Ethics Committees was not required by Danish law, due to the data being registry-based. Approval to transcribe all calls to 1-1-2 and MH-1813 was given by the Direction of the CEMS. All electronic records were anonymised before analysis, and calls were not inspected manually by the researchers.

Note that full information on the approval of the study protocol must also be provided in the manuscript.

Field-specific reporting

Please select the one below that is the best fit for your research. If you are not sure, read the appropriate sections before making your selection.

☒ Life sciences ☐ Behavioural & social sciences ☐ Ecological, evolutionary & environmental sciences

For a reference copy of the document with all sections, see nature.com/documents/nr-reporting-summary-flat.pdf

Life sciences study design

All studies must disclose on these points even when the disclosure is negative.

Sample size	The sample size for our study (N = 2,277,861) is reported in the manuscript. As we used all available data, there was no calculation to determine the sample size.
Data exclusions	We excluded registry data entries ("calls") without a diagnostic category code from the test set to ensure a valid comparison of call-takers and the machine learning framework. However, these entries were still included for testing as a separate group to assess any potential bias introduced by the exclusion. We excluded all calls that could not be linked to a corresponding CAD record as these could not be linked to the DanStroke stroke registry due to missing Danish civil registration number (CPR-number).
Replication	All model initialisations and dataset splits were drawn randomly using a seeded random number generator and are therefore reproducible. For this reason, model training and results are also reproducible. Since the data was registry-based we did not partake in the data collection process.
Randomization	We used all available registry data for the study. Subgroups of stroke-positives/negatives, age, and sex were defined on available demographic metadata and used for stratified sampling of training and validation sets for the machine learning model. The test set was not randomised but chosen as the entire available data for the year 2021.
Blinding	Blinding was not applicable to our study.

Reporting for specific materials, systems and methods

We require information from authors about some types of materials, experimental systems and methods used in many studies. Here, indicate whether each material, system or method listed is relevant to your study. If you are not sure if a list item applies to your research, read the appropriate section before selecting a response.

Materials & experimental systems

n/a	Involved in the study
☒	☐ Antibodies
☒	☐ Eukaryotic cell lines
☒	☐ Palaeontology and archaeology
☒	☐ Animals and other organisms
☒	☐ Clinical data
☒	☐ Dual use research of concern
☒	☐ Plants

Methods

n/a	Involved in the study
☒	☐ ChIP-seq
☒	☐ Flow cytometry
☒	☐ MRI-based neuroimaging
